# Estimation of unsteady hydromagnetic Williamson fluid flow in a radiative surface through numerical and artificial neural network modeling

**DOI:** 10.1038/s41598-021-93790-9

**Published:** 2021-07-15

**Authors:** Anum Shafiq, Andaç Batur Çolak, Tabassum Naz Sindhu, Qasem M. Al-Mdallal, T. Abdeljawad

**Affiliations:** 1 School of Mathematics and Statistics, Nanjing University of Information Science and Technology, Nanjing, 210044 China; 2 Mechanical Engineering Department, Niğde Ömer Halisdemir University, Niğde, Turkey; 3 Department of Statistics, Quaid- i- Azam University 45320, Islamabad, 44000 Pakistan; 4 Department of Mathematical Sciences, UAE University, P.O. Box 15551, Al-Ain, United Arab Emirates; 5 Department of Mathematics and General Sciences, Prince Sultan University, Riyadh, Saudi Arabia

**Keywords:** Mathematics and computing, Applied mathematics

## Abstract

In current investigation, a novel implementation of intelligent numerical computing solver based on multi-layer perceptron (MLP) feed-forward back-propagation artificial neural networks (ANN) with the Levenberg–Marquard algorithm is provided to interpret heat generation/absorption and radiation phenomenon in unsteady electrically conducting Williamson liquid flow along porous stretching surface. Heat phenomenon is investigated by taking convective boundary condition along with both velocity and thermal slip phenomena. The original nonlinear coupled PDEs representing the fluidic model are transformed to an analogous nonlinear ODEs system via incorporating appropriate transformations. A data set for proposed MLP-ANN is generated for various scenarios of fluidic model by variation of involved pertinent parameters via Galerkin weighted residual method (GWRM). In order to predict the (MLP) values, a multi-layer perceptron (MLP) artificial neural network (ANN) has been developed. There are 10 neurons in hidden layer of feed forward (FF) back propagation (BP) network model. The predictive performance of ANN model has been analyzed by comparing the results obtained from the ANN model using Levenberg-Marquard algorithm as the training algorithm with the target values. When the obtained Mean Square Error (MSE), Coefficient of Determination (R) and error rate values have been analyzed, it has been concluded that the ANN model can predict SFC and NN values with high accuracy. According to the findings of current analysis, ANN approach is accurate, effective and conveniently applicable for simulating the slip flow of Williamson fluid towards the stretching plate with heat generation/absorption. The obtained results showed that ANNs are an ideal tool that can be used to predict Skin Friction Coefficients and Nusselt Number values.

## Introduction

The importance of non-Newtonian substances in variety of mechanical, chemical processes and implementations in engineering is quite evident. The uses of these substances are significant in medicines, surfactants, petroleum engineering, blood and many other. However thoroughly analyzing the subgroups of non-Newtonian liquids, Williamson liquid is also categorized into these substances owing to classical characteristics of shear thinning/thickening. Having these distinct motivations in mind, several researchers adopt this model with different flow aspects and configurations^1–7^.

Thermal radiation performs a pivotal part in engineering and physics particularly in high temperature process and space technology. Most of these uses contain gas turbines, the polymer manufacturing industry, nuclear power plants and different propulsion systems for rocket, spacecraft, aircraft and satellite. Hashim et al.^8^ concentrated on radiation impacts on Williamson liquid owing to an expanding/contracting cylinder containing nanomaterials. In^9^, the impact of non-linear radiation on time dependent flow of a Williamson liquid via heat source/sink was explored. The MHD boundary layer (BL), chemical reacting and heat generating Nano-fluidic flow towards a moving radiative wedge examined in^10^. Hayat et al.^11^ explored the hydromagnetic boundary layer flow of Williamson liquid under the influence of Ohmic dissipation and radiation. For more details one can read the suggested reference^12–17^.

Magnetic fields exist anywhere in nature, so magnetohydrodynamic (MHD) mechanisms must arise when liquid conduction is accessible. It also has several engineering uses like aeronautics field, stellar/planetary magnetospheres, cosmic fluid dynamics, solar physics, MHD generators, chemical engineering, electronics, construction of turbines, MHD accelerators and many more. Whenever a magnetic field is added to an electrically conducting moving liquid, both electric and magnetic fields are induced. These fields communicate among each other, generating a body force identified as the Lorentz force, that slows down fluid movement. Recently numerous sleuths^18–24^ investigated on MHD by different fluid flows. In several practical uses, like non-mechanical MHD micropumps, the analysis of Magnetohydrodynamic slip flow demonstrated favourable performance. Reza-E-Rabbi et al.^25^ detailed the heat and mass transfer analysis of Casson nanofluid flow passing through a stretching layer with magnetohydrodynamic (MHD), thermal radiation, and chemical reaction effects. Boundary layer approximations formed the main equations, namely the momentum, energy and diffusion equilibrium equations with respect to time. The effect of various physical parameters on the momentum and thermal boundary layers is discussed and graphically illustrated together with the concentration profiles. Arifuzzaman et al.^26^ analyzed the heat and mass transfer properties of the natural convective hydromagnetic flow of the fluid with fourth order radiation originating from the vertical porous plate. The impression of heat generation by nonlinear sequential chemical reaction and thermal diffusion is also taken into account. The combined fundamental equations are transformed into a dimensionless arrangement by explicitly applying the finite difference scheme. As a result of the study, it was stated that the velocity fields started to decrease as the temperature of the fluid increased, but the opposite situation emerged for the temperature fields. Arifuzzaman et al.^27^ analyzed the appearance of nano-sized particles and the hydrodynamic flow behavior of Casson and Maxwell fluids with multiphase radiation. First, the time-dependent governing equations are solved computationally using finite difference discretization methods, and then convergence analysis is performed with the stabilization of the numerical approach. Finally, impressions of various relevant parameters are schematically depicted along with tabular analysis over diversified flow fields. The thermal and bulk properties found are significantly improved mostly in the case of Maxwell fluid. For numerical validation, some comparisons with previous studies were also shown and satisfactory agreement was observed. A partial slip would be utilized for stationary as well as moving boundary whenever a particulate liquid has been employed e.g. suspensions, emulsions, polymer solutions and foams. Several investigators have studied the significance of slip velocity influence in various flow types, including^28–31^. The hydromagnetic BL slip flow of a Maxwell nanoliquid over an exponentially expanding surface under convective boundary condition was examined by Reddy et al.^32^. The consequences of multiple slips on the flow of magneto-Carreau liquid over the wedge with chemically reactive species were studied by Khan and Hashim^33^ and the increase in shear stress and fluid velocity was investigated by increasing the magnetic parameter whereas decreasing the temperature and concentration fields.

To the best of researchers’ information, no studies has yet been made to examine the electro-hydrodynamic slip flow of Williamson fluid towards a permeable stretched surface with heat generation/absorption via ANN model. The literature summary demonstrates that in providing solutions to nonlinear issues, the ANN models have been very effective. Thus, the novelty of current study centered on usefulness of ANN procedure for boundary layer slip flow (BLSF) of Williamson fluid flow towards a stretching sheet by taking convective boundary condition and heat absorption/generation. The influences of related parameters on features of flow and heat transport are evaluated in this analysis and numerical outcomes are given in connection with the outcomes of ANN procedure.

Present study has structured as given: Section 2 includes mathematical problem formulation. Galerkin weighted residual method (GWRM) is given in Section 3. Section 4 includes the significance of ANN technique and BPA (Back Propagation algorithm). Section 5 deals with the result and discussion and Section 6 ends up with final findings .

## Problem development

**Figure 1:**
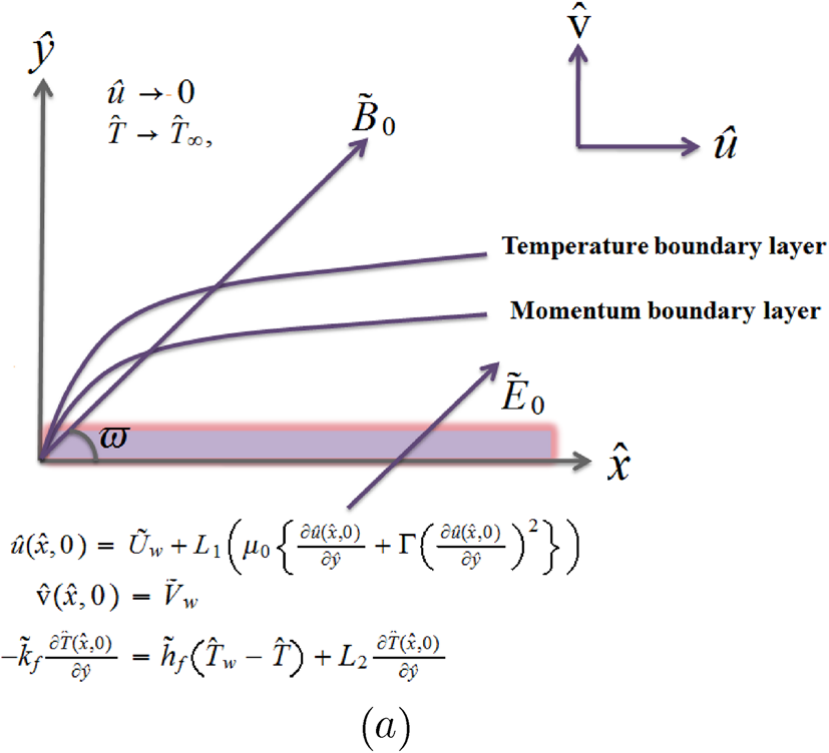
Physical configuration of the flow model.

Two dimensional incompressible unsteady electrically conducting BLF of Williamson liquid towards a porous stretched surface under velocity as well as thermal slip condition is considered. The x-axis is considered towards extending surface in direction of movement whereas y-axis is taking perpendicular as shown in Fig. 1a. The current fluidic system also incorporates viscous dissipation, heat source/sink and radiation effects. The flow area is displayed by considering uniform transverse magnetic $\bar{{\mathbf {B}}}$ and electric $\bar{{\mathbf {E}}}$ fields and known as the electrically conducting fluid. Remember that magnetic field is poorer than electric field and magnetic field follows $\bar{{\mathbf {J}}=}\sigma \left( \bar{{\mathbf {E}}}+\bar{{\mathbf {V}}}\times \bar{{\mathbf {B}}}\right)$ Ohm’s law, where $\bar{{\mathbf {J}}}$ represnts Joule current, $\sigma$ represents electrical conductivity and $\bar{{\mathbf {V}}}$ represents fluid velocity. The related flow equations while obtaining BL approximations takes the following form

1 $$\begin{aligned} \frac{\partial {\hat{u}}}{\partial {\hat{x}}}+\frac{\partial \hat{\text {v}}}{ \partial {\hat{y}}}= &  0, \end{aligned}$$

2 $$\begin{aligned} \frac{\partial {\hat{u}}}{\partial t}+\hat{\text {v}}\frac{\partial {\hat{u}}}{ \partial {\hat{y}}}+{\hat{u}}\frac{\partial {\hat{u}}}{\partial {\hat{x}}}= &  \nu \frac{ \partial ^{2}{\hat{u}}}{\partial {\hat{y}}^{2}}+2\nu {\tilde{\Gamma }}\frac{ \partial {\hat{u}}}{\partial {\hat{y}}}\frac{\partial ^{2}{\hat{u}}}{\partial \hat{ y}^{2}}+\frac{{\tilde{\sigma }}}{{\tilde{\rho }}_{0}^{*}}\sin ^{2}\left( \varpi \right) \left( {\tilde{E}}_{0}{\tilde{B}}_{0}-{\tilde{B}}_{0}^{2}{\hat{u}} \right) , \end{aligned}$$

3 $$\begin{aligned} {\tilde{\rho }}_{0}^{*}\text { }{\tilde{c}}_{p}\left( \frac{\partial {\hat{T}}}{ \partial t}+{\hat{u}}\frac{\partial {\hat{T}}}{\partial {\hat{x}}}+\hat{\text {v}} \frac{\partial {\hat{T}}}{\partial {\hat{y}}}\right)= &  {\tilde{K}}\frac{\partial ^{2}{\hat{T}}}{\partial {\hat{y}}^{2}}+{\tilde{\mu }}_{0}^{*}\left( \frac{ \partial {\hat{u}}}{\partial {\hat{y}}}\right) ^{2}+{\tilde{\mu }}_{0}^{*} {\tilde{\Gamma }}\left( \frac{\partial {\hat{u}}}{\partial {\hat{y}}}\right) ^{3}+ {\tilde{\sigma }}\sin ^{2}\left( \varpi \right) \left( {\hat{u}}{\tilde{B}}_{0}- {\tilde{E}}_{0}\right) ^{2} \nonumber \\&-\frac{\partial {\tilde{q}}_{r}^{*}}{\partial {\hat{y}}}+{\tilde{Q}} _{0}\left( {\hat{T}}-{\hat{T}}_{\infty }\right) , \end{aligned}$$

Here velocity components ${\hat{u}}$ and v̂ are in ${\hat{x}}$ and ${\hat{y}}$ directions respectively, fluid density is ${\tilde{\rho }}_{0}^{*}$, thermal conductivity is ${\tilde{K}}$, fluid temperature is ${\hat{T}}$, specific heat is ${\tilde{c}}_{p}$, kinematic viscosity is $\nu$, time constant is ${\tilde{\Gamma }}$, angel of inclination is $\varpi$ and radiative heat flux is ${\tilde{q}}_{r}^{*}$. By using the approximation of Rosseland, we have

4 $$\begin{aligned} {\tilde{q}}_{r}^{*}=-\frac{4{\check{\sigma }}^{*}}{3{\check{k}}_{1}}\frac{ \partial {\hat{T}}^{4}}{\partial {\hat{y}}}, \end{aligned}$$

in which ${\check{\sigma }}^{*}$ defines Stefan-Boltzmann constant and ${\check{k}}_{1}$ defines mean absorption coefficient. Employing Taylor’s series approximation, ${\hat{T}}^{4}\cong 4{\hat{T}}_{\infty }^{3}{\hat{T}}-3\hat{T }_{\infty }^{4}$, where ambient temperature is ${\hat{T}}_{\infty }$ and Eq. (3) becomes

5 $$\begin{aligned} {\tilde{\rho }}_{0}^{*}\text { }{\tilde{c}}_{p}\left( \frac{\partial {\hat{T}}}{ \partial t}+\hat{\text {v}}\frac{\partial {\hat{T}}}{\partial {\hat{y}}}+{\hat{u}} \frac{\partial {\hat{T}}}{\partial {\hat{x}}}\right)= &  \left( \frac{16\check{ \sigma }^{*}{\hat{T}}_{\infty }^{3}}{3{\tilde{k}}_{1}}+{\tilde{K}}\right) \frac{ \partial ^{2}{\hat{T}}}{\partial {\hat{y}}^{2}}+\mu _{0}\left( \frac{\partial {\hat{u}}}{\partial {\hat{y}}}\right) ^{2}+\mu _{0}\Gamma \left( \frac{\partial {\hat{u}}}{\partial {\hat{y}}}\right) ^{3} \nonumber \\&+{\tilde{\sigma }}\sin ^{2}\left( \varpi \right) \left( {\hat{u}}{\tilde{B}}_{0}- {\tilde{E}}_{0}\right) ^{2}+{\tilde{Q}}_{0}\left( {\hat{T}}-{\hat{T}}_{\infty }\right) . \end{aligned}$$

with

6 $$\begin{aligned} {\hat{u}}\left( {\hat{x}},0\right)= &  L_{1}\left( \mu _{0}\left\{ \frac{\partial {\hat{u}}\left( {\hat{x}},0\right) }{\partial {\hat{y}}}+\Gamma \left( \frac{ \partial {\hat{u}}\left( {\hat{x}},0\right) }{\partial {\hat{y}}}\right) ^{2}\right\} \right) +{\tilde{U}}_{w}, \nonumber \\ \hat{\text {v}}\left( {\hat{x}},0\right)= &  {\tilde{V}}_{w}=-\frac{\nu _{0}}{\left( 1-c_{1}t\right) ^{1/2}},{ \ }\left. -{\tilde{k}}_{f}\frac{\partial {\hat{T}} ({\hat{x}},0)}{\partial {\hat{y}}}={\tilde{h}}_{f}\left( {\hat{T}}_{w}-{\hat{T}} \right) +L_{2}\frac{\partial {\hat{T}}({\hat{x}},0)}{\partial {\hat{y}}},{ \ } \right. \nonumber \\&\left. {\hat{u}}\left( {\hat{x}},{\hat{y}}\rightarrow \infty \right) \rightarrow 0,{ \ \ }{\hat{T}}\left( {\hat{x}},{\hat{y}}\rightarrow \infty \right) \rightarrow {\hat{T}}_{\infty }\right. , \end{aligned}$$

and $${\tilde{V}}_{{\tilde{w}}}$$ is

7 $$\begin{aligned} {\tilde{V}}_{{\tilde{w}}}=-\frac{\nu _{0}}{\left( 1-c_{1}t\right) ^{1/2}}. \end{aligned}$$

This defines the mass transport on the surface including suction/injection $\left( {\tilde{V}}_{{\tilde{w}}}>0/{\tilde{V}}_{{\tilde{w}}}<0\right)$. Furthermore, ${\tilde{U}}_{w}\left( {\hat{x}},t\right)$ is variable stretching velocity and ${\hat{T}}_{w}\left( {\hat{x}},t\right)$ is variable wall temperature are as follows

8 $$\begin{aligned} {\tilde{U}}_{w}\left( {\hat{x}},t\right) =\frac{a_{1}{\hat{x}}}{1-c_{1}t},{ \ \ }{\hat{T}}_{w}\left( {\hat{x}},t\right) ={\hat{T}}_{\infty }+{\hat{T}}_{0}\frac{ a_{1}{\hat{x}}}{2\nu \left( 1-c_{1}t\right) ^{2}}, \end{aligned}$$

where rate constants are $a_{1}$ and $c_{1}$ with $a_{1}>0$ and $c_{1}\ge 0$ (i.e. $c_{1}t<1$). Appropriate transformation are considered as

9 $$\begin{aligned} \xi =\sqrt{\frac{{\tilde{U}}_{w}}{{\hat{x}}\nu }}y,\text { }\psi =\sqrt{\nu \hat{x }{\tilde{U}}_{w}}F\left( \xi \right) ,{ \ }\theta \left( \xi \right) = \frac{{\hat{T}}-{\hat{T}}_{\infty }}{{\hat{T}}_{w}-{\hat{T}}_{\infty }}, \end{aligned}$$

with

10 $$\begin{aligned} {\hat{u}}=\frac{\partial \psi }{\partial {\hat{y}}}, \, \hat{\text {v}}=-\frac{ \partial \psi }{\partial {\hat{x}}}. \end{aligned}$$

Eq. (1) is identically satisfied and Eqs. (2), (5) and (6) becomes

11 $$\begin{aligned}  & F^{\prime \prime \prime }-F^{\prime 2}+FF^{\prime \prime }-S_{1}\left\{ F^{\prime }+\frac{1}{2}\xi F^{\prime \prime }\right\} +2W_{e}\text { } F^{\prime \prime }F^{\prime \prime \prime }+M_{1}^{2}\sin ^{2}\left( \varpi \right) \left\{ E_{1}-F^{\prime }\right\} =0, \end{aligned}$$

12 $$\begin{aligned}&\left. \left( 1+\frac{4}{3}R_{1}\right) \theta ^{\prime \prime }+P_{r} \text { }E_{c}\text { }F^{\prime \prime 2}-P_{r}\text { }\left[ F^{\prime } \text { }\theta -\theta ^{\prime }\text { }F+\frac{S_{1}}{2}\left\{ \xi \text { }\theta ^{\prime }+4\text { }\theta \right\} \right] \right. \nonumber \\&\quad \left. +W_{e}\text { }P_{r}\text { }E_{c}\text { }F^{\prime \prime 3}+M_{1}^{2}\text { }P_{r}\text { }E_{c}\text { }\sin ^{2}\left( \varpi \right) \left[ F^{\prime }-E_{1}\right] ^{2}+P_{r}\text { }Q_{1}\theta =0,\right. \end{aligned}$$

13 $$\begin{aligned} F\text { }\left( 0\right)= &  A_{1},{ \ }F^{\prime }\left( 0\right) =1+\gamma _{1}\left[ F^{\prime \prime }\left( 0\right) +W_{e}\left\{ F^{\prime \prime }\left( 0\right) \right\} ^{2}\right] ,{\ \ }F^{\prime }\left( \xi \rightarrow \infty \right) \rightarrow 0, \nonumber \\ \theta ^{\prime }(0)= &  -\frac{B_{i}\left[ 1-\theta (0)\right] }{1+\alpha }, { \ \ \ }\theta (\xi \rightarrow \infty )\rightarrow 0, \end{aligned}$$

where $W_{e}=\Gamma {\tilde{U}}_{w}\sqrt{\frac{a_{1}}{\nu \left( 1-c_{1}t\right) }}$ represents Weissenberg number, $M_{1}^{2}=\frac{\sigma {\tilde{B}}_{0}^{2}\left( 1-c_{1}t\right) }{{\tilde{\rho }}_{0}^{*}\text { } a_{1}}$ represents magnetic number, $E_{1}=\frac{{\tilde{E}}_{0}\left( 1-c_{1}t\right) }{{\tilde{B}}_{0}a_{1}{\hat{x}}}$ represents local electric number, $A_{1}=\frac{\nu _{0}}{\sqrt{a\nu }}$ represents suction/injuction parameter, $S_{1}=\frac{c_{1}}{a_{1}}$ represents unsteadiness parameter, $\gamma _{1}=\mu _{0}L_{1}\sqrt{\frac{a_{1}}{\nu \left( 1-c_{1}t\right) }}$ represents velocity slip number, $R_{1}=\frac{4{\check{\sigma }}^{*}{\hat{T}}_{\infty }^{3}}{k^{*}{\tilde{K}}}$ represents radiation parameter, $P_{r}= \frac{\mu _{0}{\tilde{c}}_{p}}{{\tilde{K}}}$ represents Prandtl number, $\alpha = \frac{L_{2}}{{\tilde{k}}_{f}}$ represents the thermal slip number, $B_{i}= \frac{{\tilde{h}}_{f}}{{\tilde{k}}_{f}}\sqrt{\frac{\nu {\hat{x}}}{{\tilde{U}}_{w}}}$ defines Biot number, $Q_{1}=\frac{{\tilde{Q}}_{0}\left( 1-c_{1}t\right) }{ {\tilde{\rho }}_{0}^{*}{\tilde{c}}_{p}a_{1}}$ defines the heat generation/ absorption parameter and $E_{c}=\frac{{\tilde{U}}_{w}^{2}}{{\tilde{c}}_{p}\left( {\hat{T}}_{w}-{\hat{T}}_{\infty }\right) }$ represents the Eckert number.

Expression of skin friction coefficient is

14 $$\begin{aligned}&\left. {\tilde{C}}_{F}=\frac{\tau _{w}}{\rho ^{*}{\tilde{U}}_{w}^{2}}= \frac{\left[ \mu _{0}\left\{ \frac{\partial {\hat{u}}}{\partial {\hat{y}}} +\Gamma \left( \frac{\partial {\hat{u}}}{\partial {\hat{y}}}\right) ^{2}\right\} \right] _{{\hat{y}}\text { }=\text { }0}}{\rho ^{*}{\tilde{U}}_{w}^{2}},\right. \nonumber \\&\left. {\text {Re}}_{{\hat{x}}}^{\frac{1}{2}}{\tilde{C}}_{F}=\left[ F^{\prime \prime }\left( 0\right) +We\text { }F^{\prime \prime 2}\left( 0\right) \right] .\right. \end{aligned}$$

Expression of local Nusselt number (LNN) is

15 $$\begin{aligned}&\left. Nu_{{\hat{x}}}=\frac{{\hat{x}}\text { }q_{w}}{{\tilde{K}}\left( {\hat{T}}_{w}-{\hat{T}}_{\infty }\right) }=-\frac{{\hat{x}}\left( \frac{16\sigma ^{*} {\tilde{T}}_{\infty }^{3}}{3k_{1}}+{\tilde{K}}\right) \left. \frac{\partial {\tilde{T}}}{\partial {\hat{y}}}\right| _{{\hat{y}}\text { }=\text { }0}}{\tilde{ K}\left( {\hat{T}}_{w}-{\hat{T}}_{\infty }\right) },\right. \nonumber \\&\left. {\text {Re}}_{x}^{-1/2}Nu_{{\hat{x}}}=-\left( 1+\frac{4}{3}R_{1}\right) \theta ^{\prime }(0).\right. \end{aligned}$$

## Galerkin weighted residual method (GWRM)

GWRM is an effective method for calculating solutions of nonlinear BVP (boundary value problems). It comprises the following main steps: (1) In differential equations, the unknown dependent functions are initially considered to be linear combinations of form or trial functions containing unknown coefficients. (2) Such supposed solutions are incorporated into equations that contains residuals. (3) The errors are forced to be as small utilizing certain weight functions, therefore found unknown coefficients. The key characteristics that make this procedure (GWRM) appealing are (a) The ease of handling BVPs relating semi-infinite range. (b) It has high precision, performance and quick convergence. (c) the associated range within 0 and $\infty$ is directly minimized. Therefore, we utilized GWRM to find the solution of governing differential system (11–12) with (13). GWRM procedures to seek an approximate solution as follows

16 $$\begin{aligned} F\left( x\right) +{ {\tilde{L}}}\left( \chi \left( x\right) \right) =0 \ \ \text {in} \ \ \ { {\tilde{D}}}_{0}, \end{aligned}$$

here unknown dependent variable is $\chi \left( x\right)$, independent function is $F\left( x\right)$ in domain ${ {\tilde{D}}}_{0}$ and differential operator is ${ {\tilde{L}}}$. An approximate solution

17 $$\begin{aligned} \chi \left( x\right) =\chi _{0}+\sum _{k=1}^{n}a_{k}\chi _{k}\left( x\right) , \end{aligned}$$

is defined in fashion that it ensures the specified boundary conditions. Replacing Eq. (17) into Eq. (16) emanated in ${\tilde{R}}\left( x\right) . {\tilde{R}}\left( x\right)$ (residual function) is reduced as little as possible in ${ {\tilde{D}}}_{0}$ by putting the integral of product of $\chi _{k}\left( x\right)$ (weight functions) and ${\tilde{R}}\left( x\right)$ over whole ${ {\tilde{D}}}_{0}$ equal to zero for $k\ge 0,$ *n*.

18 $$\begin{aligned} \int _{{ D}}{\tilde{R}}\left( x\right) \chi _{k}\left( x\right) dx=0, { \ \ \ }k=0,1,...,n. \end{aligned}$$

Gauss–Laguerre formula is employed to integrate every equations in (18) to achieve set of algebraic systems since boundary condition varies from zero to infinity. The $a_{k}$ values are gained via solving the consequent algebraic systems.

### Gauss–Laguerre formula (GLF)

GLF is employed as given below^34^:

19 $$\begin{aligned} \int _{0}^{\infty }e^{-x}F\left( x\right) dx\approx \sum _{k=1}^{n}B_{k}F\left( x_{k}\right) , \end{aligned}$$

here $B_{k}$ coefficients have been specified as^35^

20 $$\begin{aligned} B_{k}=\frac{1}{{ {\tilde{L}}}_{n}^{\prime }\left( x_{k}\right) } \int _{0}^{\infty }\frac{e^{-x}{ {\tilde{L}}}_{n}\left( x\right) }{x-x_{k} }dx=\frac{\left( n!\right) ^{2}}{x_{k}\left( { {\tilde{L}}}_{n}^{\prime }\left( x_{k}\right) \right) ^{2}}, \end{aligned}$$

and $x_{k}$ are the zeros of $n^{th}$ Laguerre polynomial

21 $$\begin{aligned} { {\tilde{L}}}_{n}=e^{x}\frac{d^{n}}{dx^{n}}\left[ e^{-x}x^{n}\right] . \end{aligned}$$

For $n=10,$ Table 1 displays $x_{k}$ values and relating $B_{k}$ values.

### Application of GWRM’s to current problem

Using GWRM, the assumed solutions of $F\left( \xi \right)$ and $\theta \left( \xi \right)$ are considered as given below^34^

22 $$\begin{aligned} F\left( \xi \right) =\sum \limits _{i=0}^{{\tilde{N}}}a_{i}\text { }e^{-\frac{ i\xi }{3}}, { \ \ \ }\theta \left( \xi \right) =\sum \limits _{k=1}^{ {\tilde{N}}}b_{k}\text { }e^{-\frac{k\xi }{3}}. \end{aligned}$$

Selecting ${\tilde{N}}=15,$ substituted Eq. (22) into Eq. (13), to atain

23 $$\begin{aligned}&-A+a_{0}+a_{1}+a_{2}+a_{3}+a_{4}+a_{5}+a_{6}+a_{7}+a_{8}+a_{9}+a_{10}+a_{11} \nonumber \\&\left. +a_{12}+a_{13}+a_{14}+a_{15}=0,\right. \end{aligned}$$

24 $$\begin{aligned}&-1-\frac{a_{1}}{3}-\frac{2a_{2}}{3}-a_{3}-\frac{4a_{4}}{3}-\frac{5a_{5}}{3} -2a_{6}-\frac{7a_{7}}{3}-\frac{8a_{8}}{3}-3a_{9}-\frac{10a_{10}}{3} \nonumber \\&-\frac{11a_{11}}{3}-4a_{12}-\frac{13a_{13}}{3}-\frac{14a_{14}}{3}-5a_{15}- \nonumber \\&\gamma _{1}\left[ \left( \frac{a_{1}}{9}+\frac{4a_{2}}{9}+a_{3}+\frac{ 16a_{4}}{9}+\frac{25a_{5}}{9}+4a_{6}+\frac{49a_{7}}{9}+\frac{64a_{8}}{9} +9a_{9}\right. \right. \nonumber \\&\quad \left. +\frac{100a_{10}}{9}+\frac{121a_{11}}{9}+16a_{12}+\frac{169a_{13}}{9 }+\frac{196a_{14}}{9}+25a_{15}\right) \nonumber \\&+W_{e}\left( \frac{a_{1}}{9}+\frac{4a_{2}}{9}+a_{3}+\frac{16a_{4}}{9}+ \frac{25a_{5}}{9}+4a_{6}+\frac{49a_{7}}{9}+\frac{64a_{8}}{9}+9a_{9}\right. \nonumber \\&\quad \left. \left. \left. +\frac{100a_{10}}{9}+\frac{121a_{11}}{9}+16a_{12}+ \frac{169a_{13}}{9}+\frac{196a_{14}}{9}+25a_{15}\right) ^{2}\right) =0,\right. \end{aligned}$$

25 $$\begin{aligned}&-\frac{b_{1}}{3}-\frac{2b_{2}}{3}-b_{3}-\frac{4b_{4}}{3}-\frac{5b_{5}}{3} -2b_{6}-\frac{7b_{7}}{3}-\frac{8b_{8}}{3}-3b_{9}-\frac{10b_{10}}{3} \nonumber \\&-\frac{11b_{11}}{3}-4b_{12}-\frac{13b_{13}}{3}-\frac{14b_{14}}{3}-5b_{15}+ \frac{B_{i}}{1+\alpha }\left\{ 1-b_{1}-b_{2}-b_{3}\right. \nonumber \\&\left. \left. -b_{4}-b_{5}-b_{6}-b_{7}-b_{8}-b_{9}-b_{10}-b_{11}-b_{12}-b_{13}-b_{14}-b_{15}\right\} =0.\right. \end{aligned}$$

The boundary conditions at infinity in Eq. (13) are automatically satisfied. Putting Eq. (22) into Eqs. (11) and (12) occurred in ${\tilde{R}}_{F}\left( a_{i},\xi \right)$ and ${\tilde{R}}_{\theta }\left( a_{i},b_{k},\xi \right)$ for $i=0,1,\cdots ,15,$ $k=1,2,\cdots ,15.$ First, minimize residual by taking integral of product of residual and weight functions $e^{-\frac{i\xi }{3}}$ and $e^{-\frac{k\xi }{3}}$, for $k=1,2,\ldots ,{\tilde{N}}-1,\ i=0,1,\ldots ,{\tilde{N}}-2$ to zero, *i*.*e*.

26 $$\begin{aligned} \int _{0}^{\infty }{\tilde{R}}_{F}\left( a_{i},\xi \right) \text { }e^{-\frac{ i\xi }{3}}d\xi =0,{ \ }\int _{0}^{\infty }{\tilde{R}}_{\theta }\left( a_{i},b_{k},\xi \right) \text { }e^{-\frac{k\xi }{3}}d\xi =0, \end{aligned}$$

together with Eqs. (23–25) produce $2{\tilde{N}}+1$ nonlinear algebraic systems with $2{\tilde{N}}+1$ unknown coefficients $\left( a_{i},b_{k}\right)$ and then solved via MATHEMATICA to get $a_{i}$ and $b_{k}$.

## Neural network modeling

**Figure 2:**
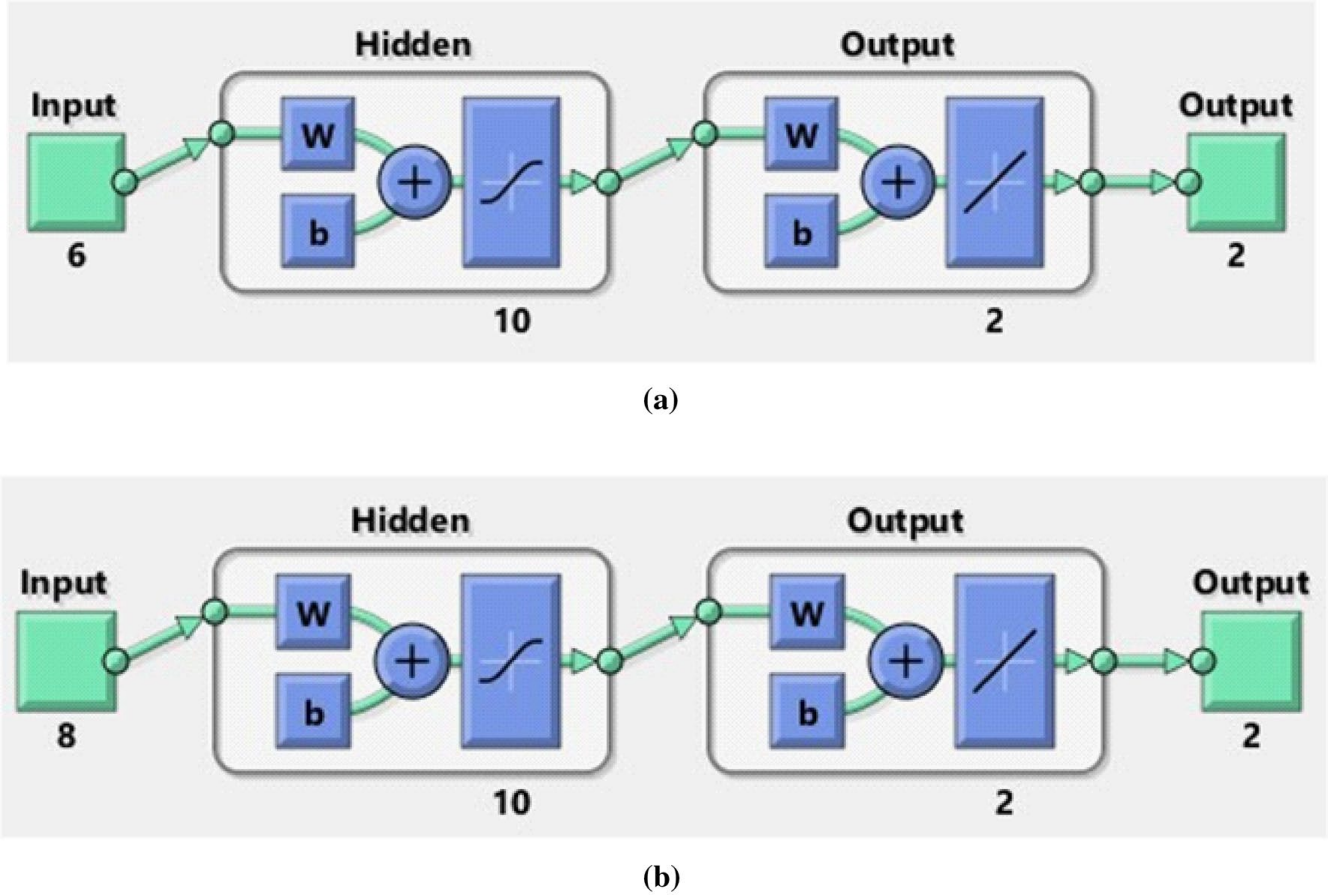
The basic structures of the ANN models (**a**) SFCs (**b**) NN.

Due to the difficulties of experimental studies, long time and cost, many researchers have worked on numerical modeling and derivation of mathematical correlations. Misidentification and modeling of experimental and theoretical data may cause errors in the results obtained from the simulation study. In addition, there are various difficulties in modeling nonlinear and non-linear mathematical functions with traditional tools^36^. Artificial neural networks (ANN), which were developed on the basis of the biological working principle of the human brain, have been one of mathematical measures that are frequently utilized by investigators^37^. ANNs started to be used in the middle of the twentieth century. They have a wide range of applications due to their fastness, flexibility, learning algorithms, and tolerance to errors^38,39^. Thanks to these important advantages, ANNs have recently become tools that are frequently used in various fields such as medicine and business as well as many different engineering applications^40–44^. One of the most frequently used models among ANN models is MLP network model, that has a feed-forward back-propagation (FFBP) structure^45,46^. An MLP network has an input layer where input parameters are defined, at least one hidden and one output layer, where predictive values are gained. The hidden layer contains processing elements called neurons, and each layer is connected to the other with a transfer function. Optimizing the data to be used in training of ANN is one of the important parameters affecting the prediction accuracy of ANN. For this reason, the data used in ANN models should be grouped and optimized ideally^47^. In this study, two different ANN models have been designed in order to predict SFC and NN. The data set used in both ANN models is divided into three parts, which are frequently preferred by the researchers. $$70\%$$ of the data have been used for training, $$15\%$$ for validation and $$15\%$$ for testing^48^. In the input layer of the ANN model designed for SFC prediction, $$ W_{e},$$ *A*,  $$S_{1},$$ $$M_{1},$$ $$E_{1}$$ and $$\gamma _{1}$$ values have been defined as input parameters, and the skin friction coefficient value has been predicted at output layer. In MLP network model, which has been designed with a total of 28 data sets, 20 of data have been used for the training phase, 4 for the validation phase and 4 for the test phase. In ANN model developed for prediction of NN; $$M_{1},$$ $$E_{1},$$ $$R_{1},$$ $$P_{r}$$, $$ E_{c},$$ $$Q_{1},$$ $$B_{i}$$ and $$\alpha $$ values are defined as input parameters and Nusselt Number is predicted at output layer. In ANN model using a total of 33 data sets, 25 of data have been used for training, 5 for validation, and 5 for testing. There is no exact methodology for determining the number of neurons to be used in ANNs^49^. For this reason, both ANN models have been developed with different neuron numbers and their performances have been analyzed. By evaluating the obtained results, 10 neurons have been used in the hidden layers of both ANN models. The basic structures of the ANN models developed are shown in Fig. 2a,b.

In the developed ANN models, the Levenberg–Marquardt algorithm, which is one of the powerful algorithms widely preferred in the literature, has been used as the training algorithm^50^. In the hidden layer of ANN models, Tan-Sig function is used as the transfer function and Purelin functions in the output layer^51^. The transfer functions utilized is provided as:

27 $$\begin{aligned} {\tilde{f}}\left( x\right)= &  \frac{1}{1+e^{-x}}, \end{aligned}$$

28 $$\begin{aligned} \text {purelin}(x)= &  x, \end{aligned}$$

Mean Square Error (MSE), Coefficient of Determination (R) parameters have been used for performance analysis of the developed MLP network model. In addition, the error rates between values attained from ANN model and the target values have also been calculated and analyzed. The equations used in the calculation of performance parameters are given below^52^:

29 $$\begin{aligned} MSE= &  \frac{1}{N}\sum _{i=1}^{N}\left( X_{\exp \left( i\right) }-X_{ANN\left( i\right) }\right) ^{2}, \end{aligned}$$

30 $$\begin{aligned} R= &  \sqrt{1-\frac{\sum _{i=1}^{N}\left( X_{\exp \left( i\right) }-X_{ANN\left( i\right) }\right) ^{2}}{\sum _{i=1}^{N}\left( X_{\exp \left( i\right) }\right) ^{2}},} \end{aligned}$$

31 $$\begin{aligned} \text {Error Rate}\left( \%\right)= &  \left[ \frac{X_{\exp }-X_{ANN}}{X_{\exp }} \right] \times 100. \end{aligned}$$

## Results and discussion

**Figure 3:**
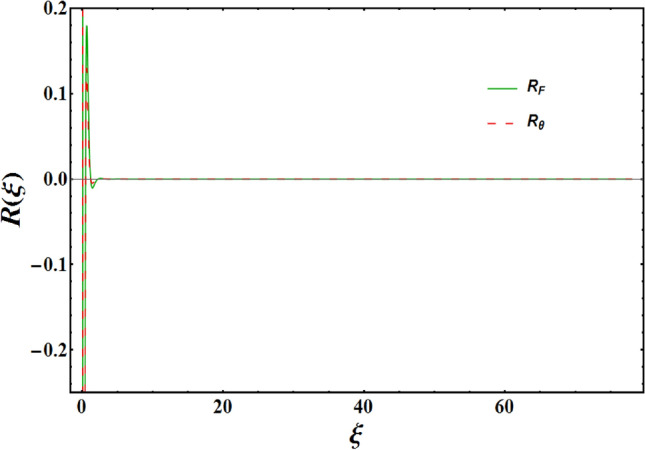
Minimized redidual error $\left( {\tilde{R}}_{F},{\tilde{R}}_{\theta }\right)$.

**Figure 4:**
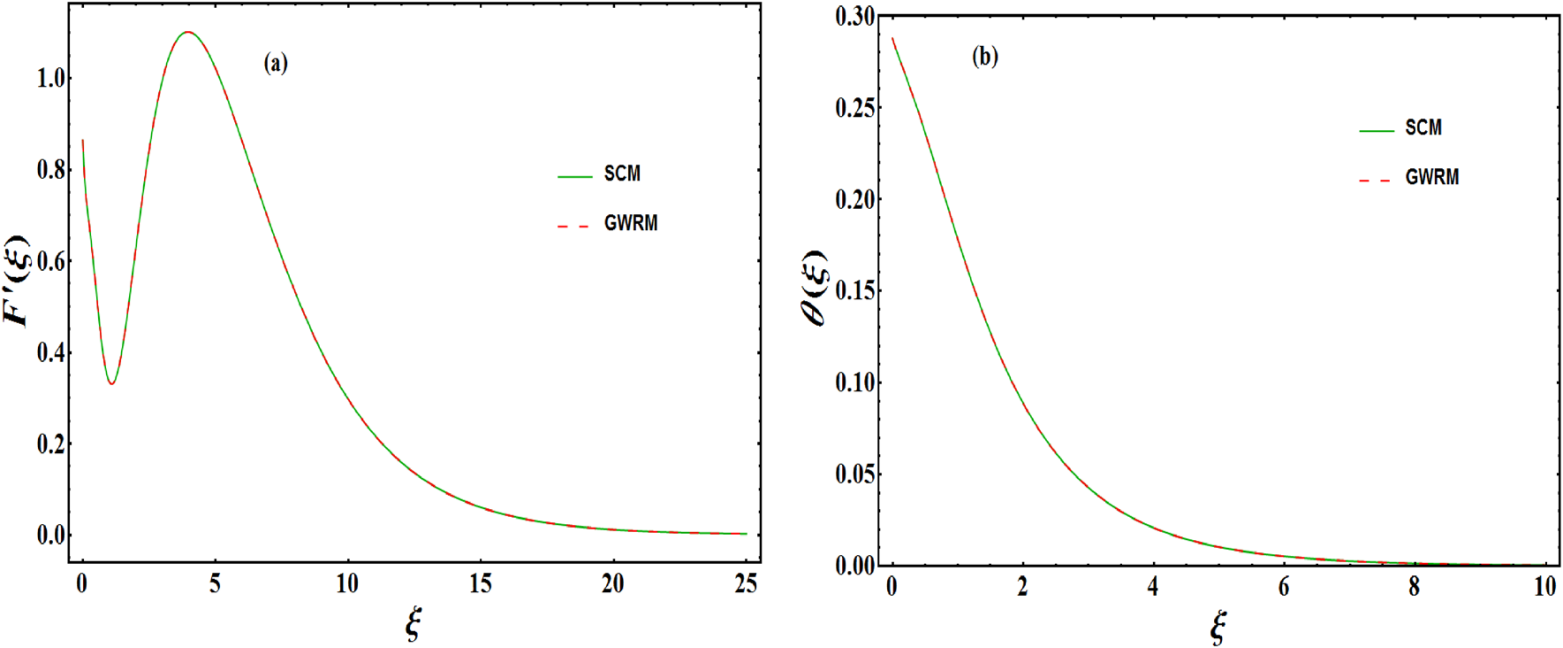
Comparison of the velocity and temperature profiles attained via SCM and GWRM.

**Figure 5:**
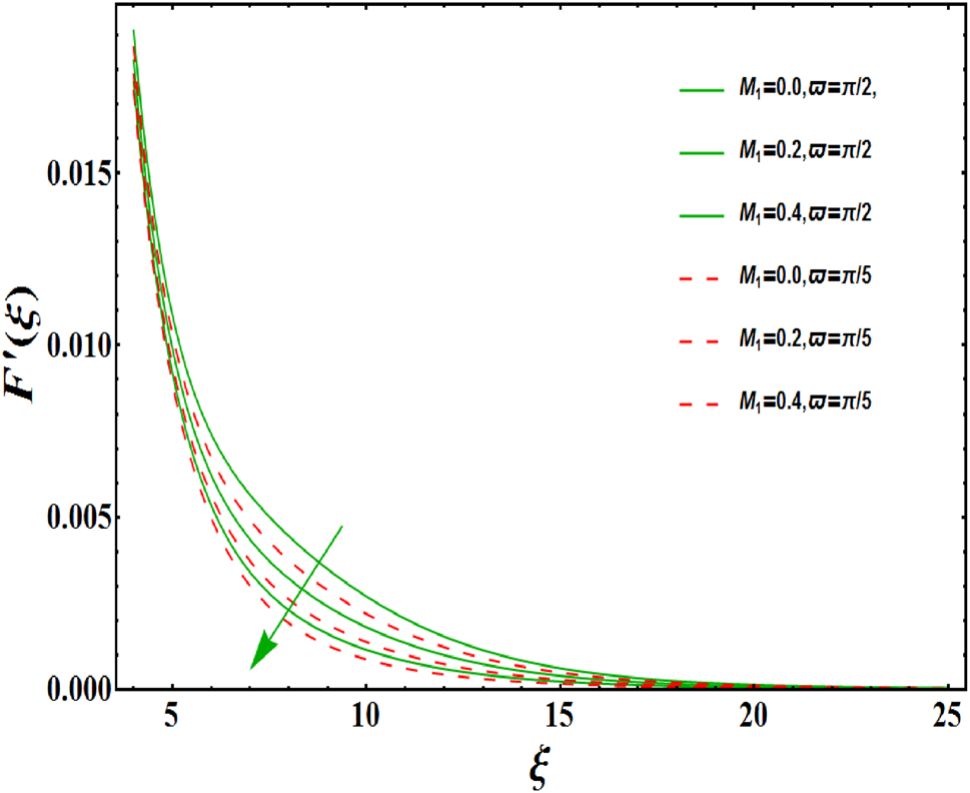
Variation of $M_{1}$ on $F^{\prime }\left( \xi \right)$ for both with and without inclanation.

**Figure 6:**
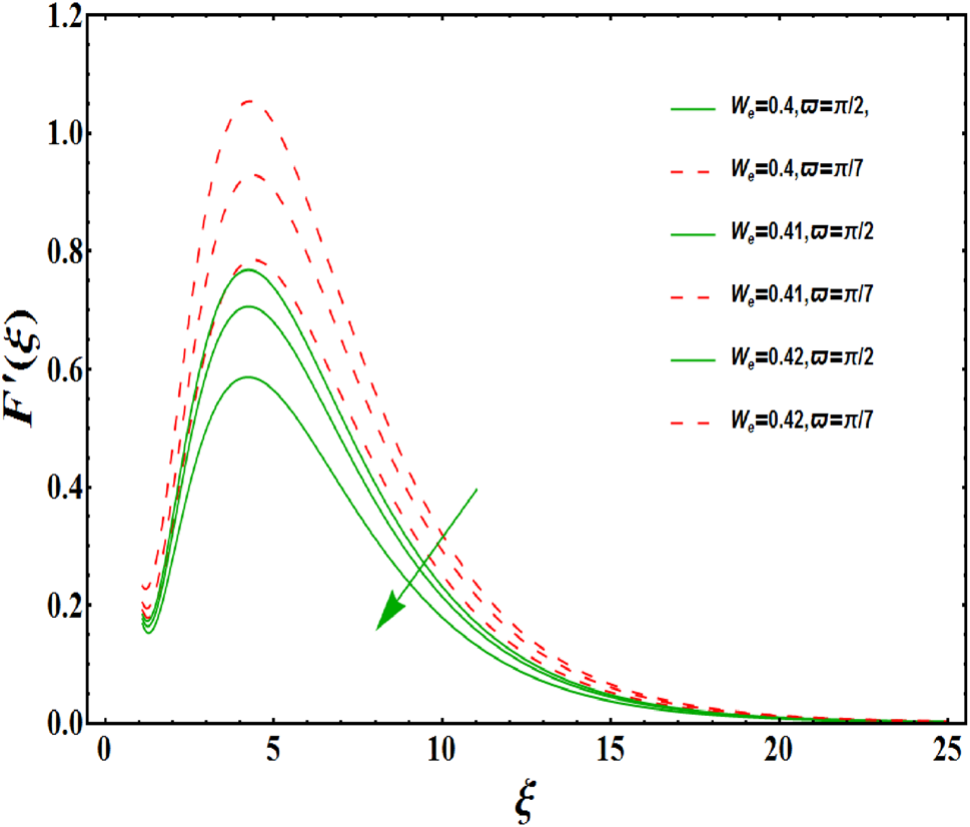
Variation of $W_{e}$ on $F^{\prime }\left( \xi \right)$ for both with and without inclanation.

**Figure 7:**
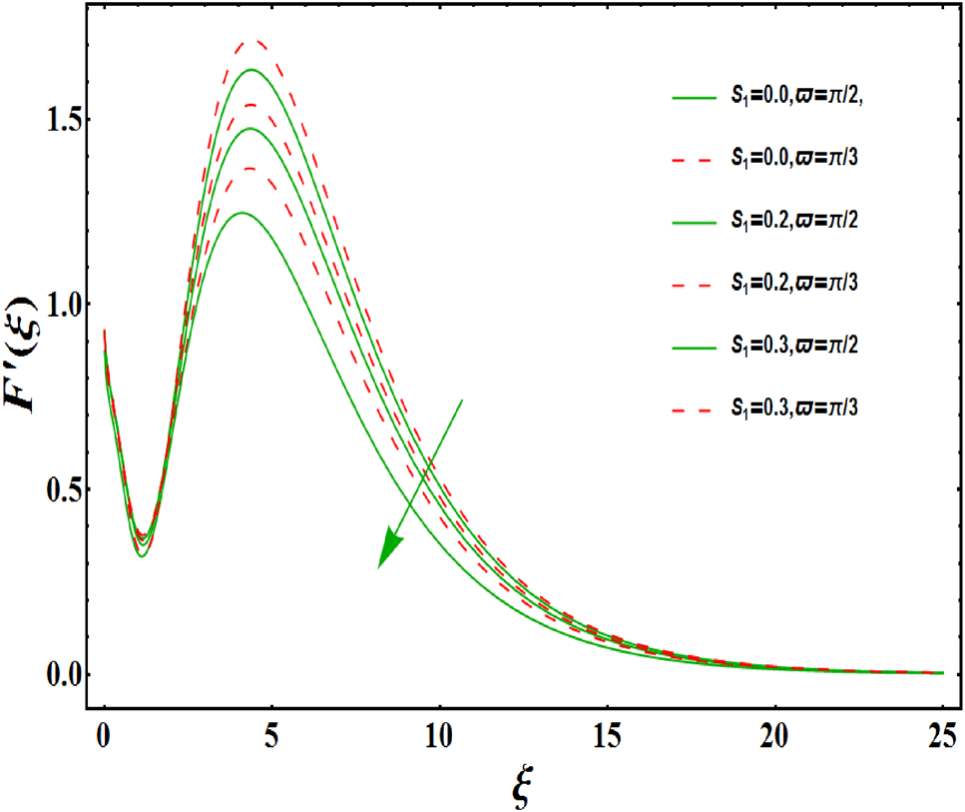
Variation of $S_{1}$ on $F^{\prime }\left( \xi \right)$ for both with and without inclanation.

**Figure 8:**
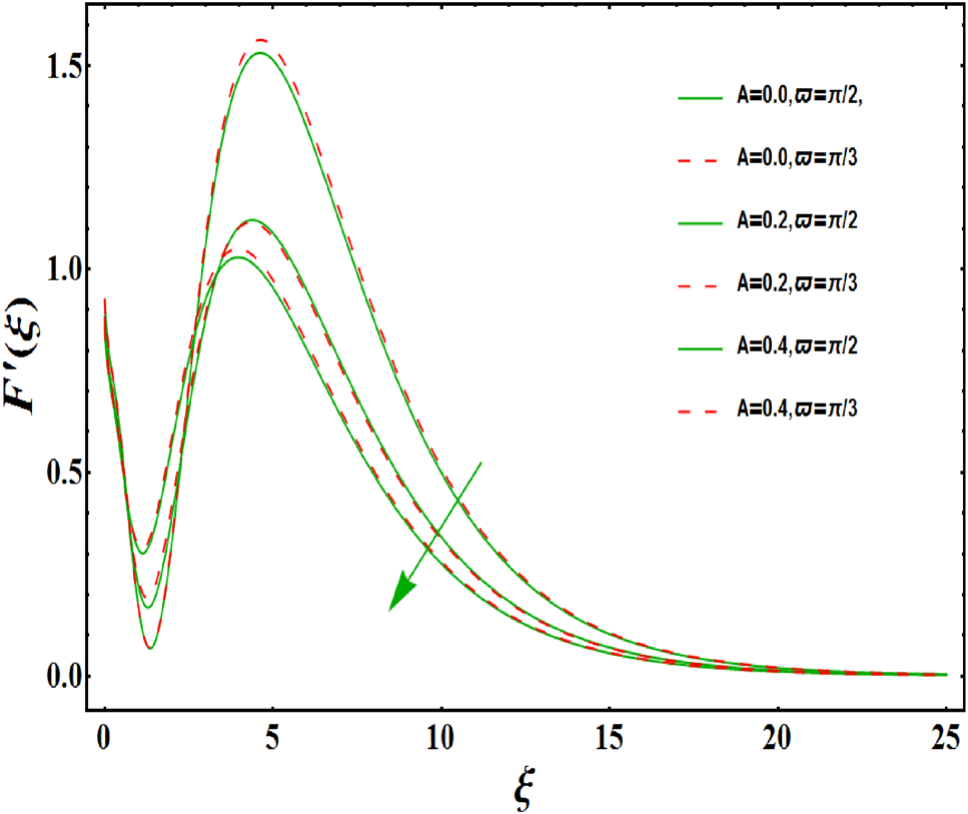
Variation of $A>0$ on $F^{\prime }\left( \xi \right)$ for both with and without inclanation.

**Figure 9:**
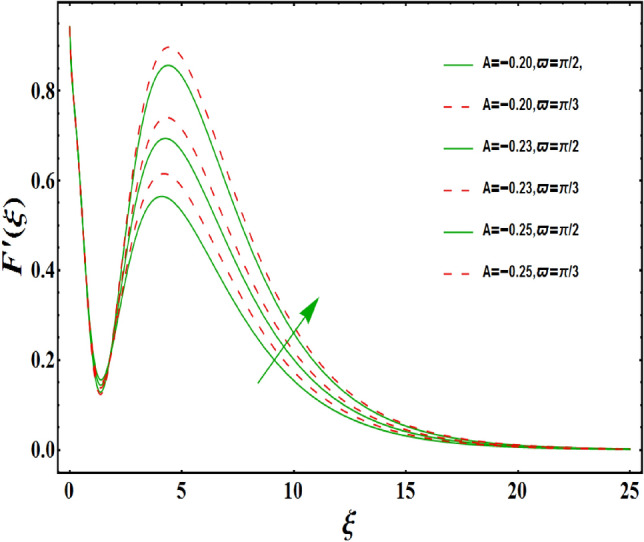
Variation of $A<0$ on $F^{\prime }\left( \xi \right)$ for both with and without inclanation.

**Figure 10:**
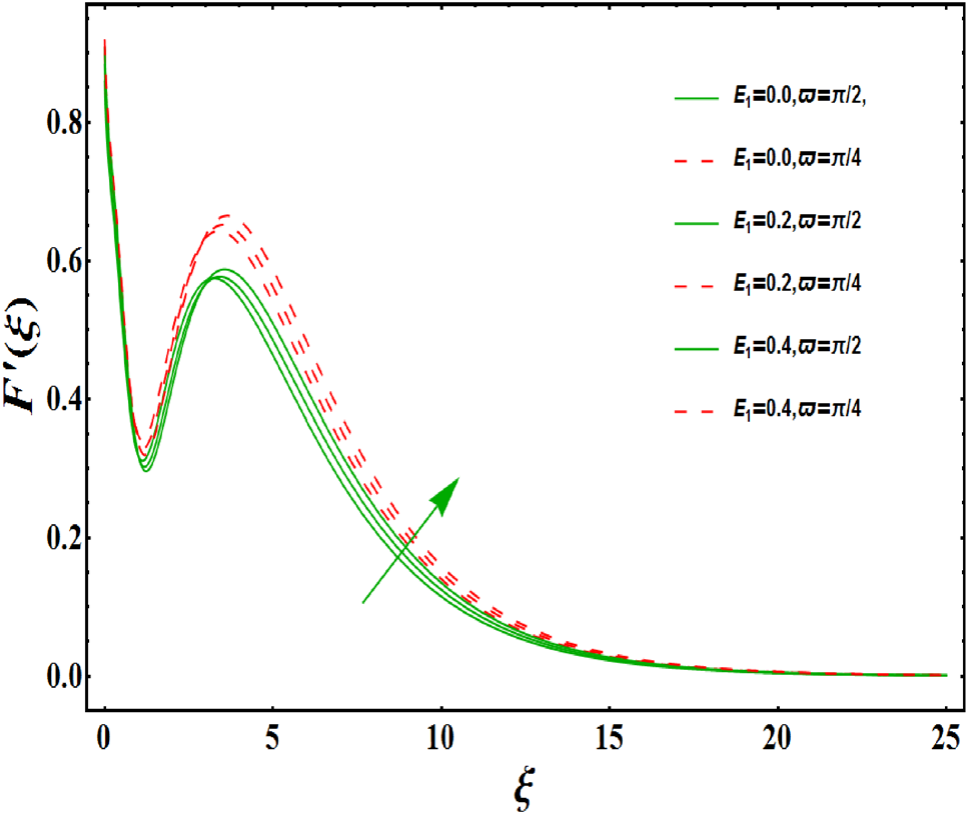
Variation of $E_{1}$ on $F^{\prime }\left( \xi \right)$ for both with and without inclanation.

**Figure 11:**
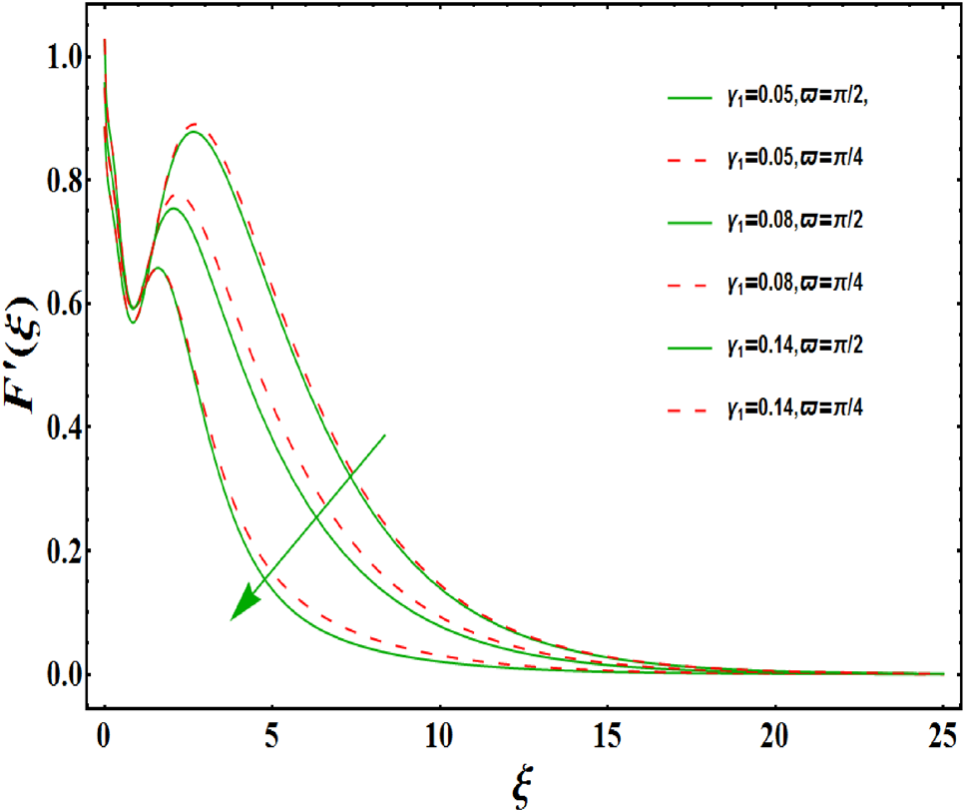
Variation of $\gamma _{1}$ on $F^{\prime }\left( \xi \right)$ for both with and without inclanation.

**Figure 12:**
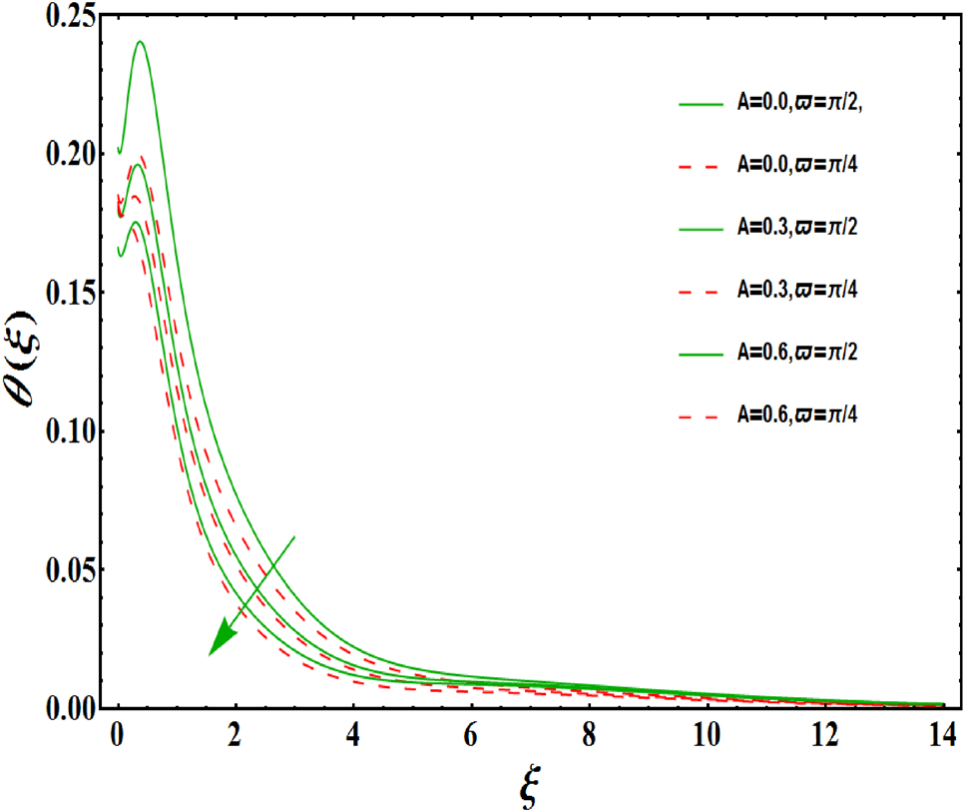
Variation of $A>0$ on $\theta \left( \xi \right)$ for both with and without inclanation.

**Figure 13:**
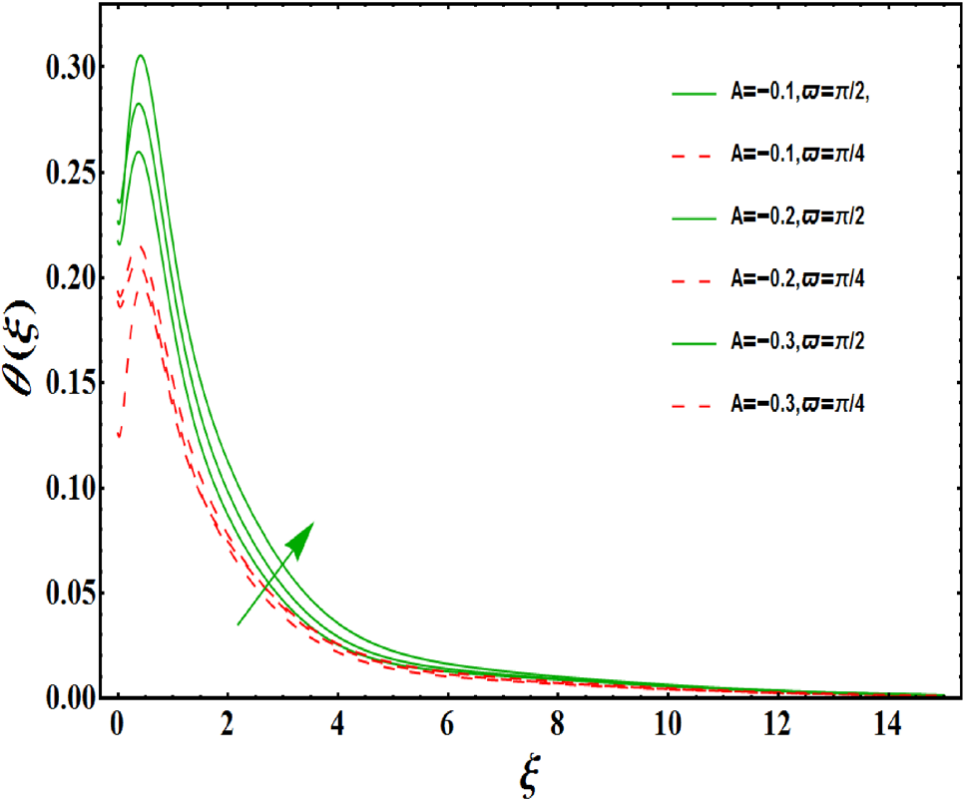
Variation of $A<0$ on $\theta \left( \xi \right)$ for both with and without inclanation.

The GWRM method has been utilized to compute numerical simulation of temperature and velocity fields within boundary layer for various values of related physical parameters. We explore structural features of all associated dimensionless parameters on velocity $$F^{\prime }\left( \xi \right) $$ and temperature $$\theta \left( \xi \right) $$ fields, that are portrayed through Figs. 5, 6, 7, 8, 9, 10, 11, 12, 13 14 and 15. For the leading variables, we gave default values as: $$A=0.4=M_{1}$$; $$S_{1}=0.3=R_{1};$$ $$Q_{1}=W_{e}=0.5=E_{c}=E_{1};$$ $$ P_{r}=1;$$ $$\gamma _{1}=\alpha =0.2=B_{i};$$ during the entire computations unless otherwise mentioned. The map of $${\tilde{R}}_{F}\left( a_{i},\xi \right) $$ and $${\tilde{R}}_{\theta }\left( a_{i},b_{k},\xi \right) $$ are displayed in Fig. 3. It is noted that residuals in the range $$(0-\infty )$$ are reduced. Further, to see the reliability of the approach employed, a simulation study is presented. This is obtained by analyzing graphical findings of dimensionless velocity and temperature by incorporating spectral collocation method (SCM) and Galerkin weighted residual method (GWRM) (see Fig. 4a,b). In each of the cases, an outstanding agreement is found.

**Figure 14:**
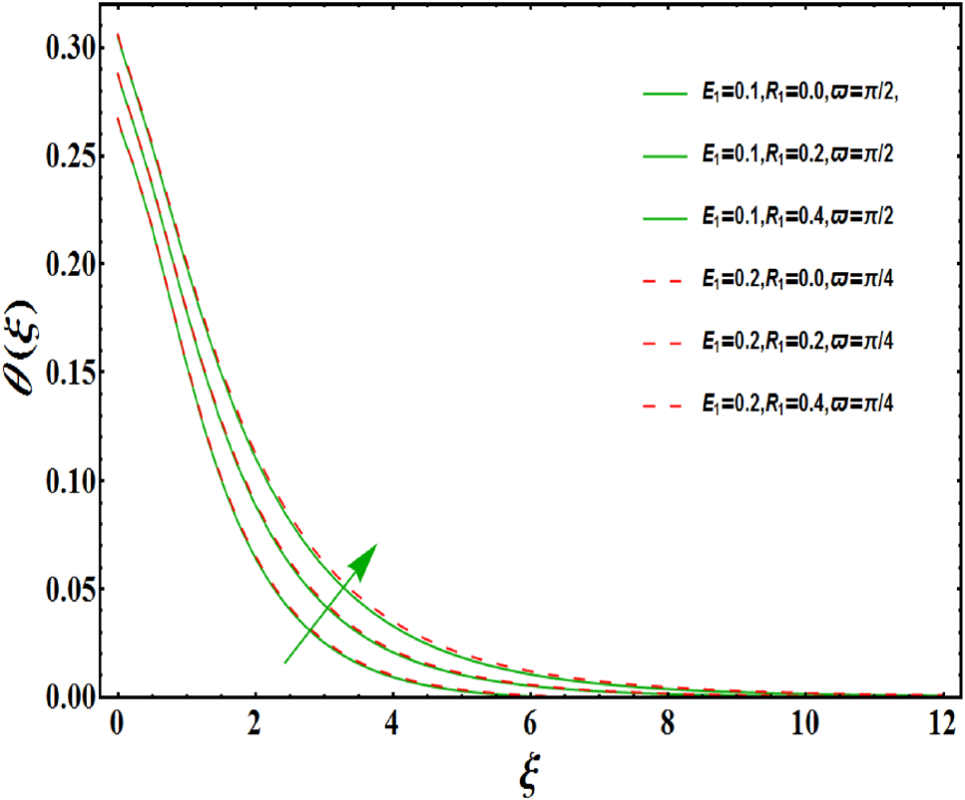
Variation of $R_{1}$ on $\theta \left( \xi \right)$ for both with and without inclanation.

**Figure 15:**
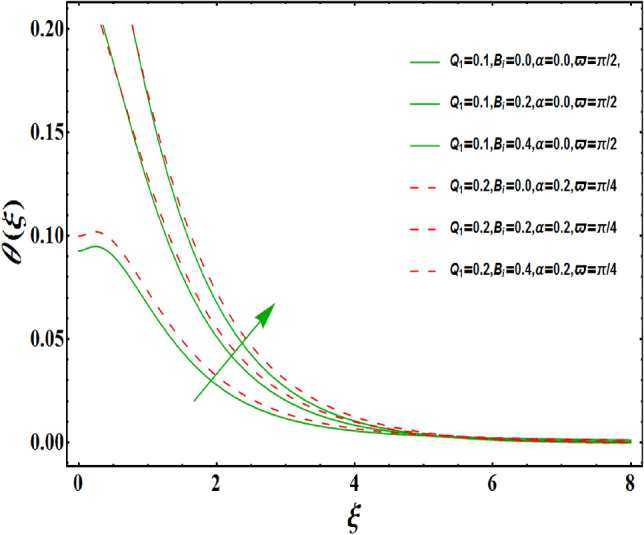
Variation of $B_{i}$ on $\theta \left( \xi \right)$ for both with and without inclanation.

The dimensionless velocity profiles $$F^{\prime }\left( \xi \right) $$ for numerous values of magnetic number $$M_{1}$$ are demonstrated in Fig. 5. The numerical values are mapped for two separate scenarios of inclination parameter $$\varpi $$ i.e., non-inclined MHD $$\left( \varpi =\pi /2\right) $$ and inclined MHD $$\left( \varpi =\pi /5\right) $$. It is observed in Fig. 5 that velocity profiles inside the boundary layer declines for higher values of $$M_{1}$$ for both situations. This statement is scientifically justified since the existence of a transverse magnetic field in an electrically conducting liquid gives rise to a Lorentz force (resistive force), that slows down the movement of liquid inside the area of BL. We also observe that, after a certain distance from the solid surface, the measured effects are very noticeable. Additionally, we notice that for the inclination scenario the velocity profile is lower than the non-inclination scenario. The influences of Weissenberg parameter $$W_{e}$$ on fluid velocity $$F^{\prime }\left( \xi \right) $$ is provided in Fig. 6 for two separate scenarios of inclination parameter $$\varpi $$ i.e., non-inclined MHD $$\left( \varpi =\pi /2\right) $$ and inclined MHD $$\left( \varpi =\pi /7\right) $$. It is remarkably noticed that the velocity fields are lessening by enhancing values of $$W_{e}$$ for both scenarios. The Weissenberg number $$W_{e}$$ provides ratio of relaxation to specific process times. Growing $$W_{e}$$ causes a reduction in specific process time that consequence will lead a decline in velocity component and BL thickness. Further, we noticed that velocity field is remarkably higher in inclined MHD case than non-inclined MHD. The finding in Fig. 7 describes that velocity profile is declining functions of unsteadiness variable in BL for both scenarios of inclination parameter $$\varpi $$ i.e., non-inclined MHD $$\left( \varpi =\pi /2\right) $$ and inclined MHD $$\left( \varpi =\pi /3\right) $$. This is owing to the belief that as unsteadiness factor $$S_{1}$$ grows, the velocity of stretched surface also reduces that further causes the conversion of less amount of heat and mass from the plate to the fluid in the boundary layer region. Figure 8 is sketched to view the distribution of fluid velocity for several values of suction parameter $$\left( A>0\right) $$ for two separate scenarios of inclination parameter $$\varpi $$ i.e., non-inclined MHD $$\left( \varpi =\pi /2\right) $$ and inclined MHD $$\left( \varpi =\pi /3\right) $$. It can be noted that when the values of the suction parameter rises, the velocity field and associated boundary layer thickness are lessened for both cases. This is bases the fact that suction or blowing is the way of controlling the boundary layer. The suction method involves extracting decelerated liquid particles from the boundary layer until they are given the opportunity to cause separation. Moreover, we noticed that velocity field is higher in inclined MHD case than non-inclined MHD. Figure 9 demonstrates the velocity field $$F^{\prime }\left( \xi \right) $$ for different values of injection $$ \left( A<0\right) $$ for both scenarios of inclination parameter $$\varpi $$ i.e., non-inclined MHD $$\left( \varpi =\pi /2\right) $$ and inclined MHD $$ \left( \varpi =\pi /3\right) $$. This figure illustrates that velocity grow with an improvement in injection parameter for both cases. Furthermore, we noted that velocity field is declines in case of non-inclined MHD $$\left( \varpi =\pi /2\right) $$ than inclined MHD $$\left( \varpi =\pi /3\right) $$. The influence of electric parameter $$E_{1}$$ on fluid velocity field $$ F^{\prime }\left( \xi \right) $$ is showed in Fig. 10 for both scenarios of inclination parameter $$\varpi $$ i.e., non-inclined MHD $$\left( \varpi =\pi /2\right) $$ and inclined MHD $$\left( \varpi =\pi /4\right) $$. We scrutinized that the related boundary layer grows at a slightly lower rate near the wall for greater values of electrical parameter $$E_{1}$$, whereas it tends to increase more dramatically away from the stretching surface for both situations. This study indicates that moving the streamlines away from the extended boundary is the consequence of electrical parameters. It is due to the Lorentz force that causes reduction in the frictional resistance. In addition, we found that velocity field is significantly higher in case of inclined MHD than non-inclined MHD for electrical parameter $$E_{1}$$. For several values of velocity slip parameter $$\gamma _{1},$$ the variability of velocity field is mapped in Fig. 11 for both case of inclination parameter $$\varpi $$ i.e., non-inclined MHD $$\left( \varpi =\pi /2\right) $$ and inclined MHD $$\left( \varpi =\pi /4\right) $$. Figure 11 clearly demonstrates that velocity curves drop significantly as value of velocity slip number rises for both cases when $$\varpi =\pi /2$$ and $$\varpi =\pi /4$$. This is owing to the belief that slip velocity grows as slip parameter rises and liquid velocity declines as the pulling of stretching wall can only be partially transmitted to the fluid under the slip condition. Figures 12 and 13 are plotted to observed the variability of temperature field $$\theta \left( \xi \right) $$ for different values of the suction/injection parameter $$\left( A>0,A<0\right) $$, respectively for both non-inclined MHD $$\left( \varpi =\pi /2\right) $$ and inclined MHD $$\left( \varpi =\pi /4\right) $$. From said figures, it can be noted that enhancement in suction parameter $$ \left( A>0\right) $$ causes the reduction in temperature and associated boundary layer thickness whereas opposite behaviour is noted for the injection $$\left( A<0\right) $$ parameter when $$\varpi =\pi /2$$ and $$\varpi =\pi /4$$. Furthermore, in case of non-inclinied MHD, temperature field is remarkably higher. The finding in Fig. 14 indicates that the temperature profile $$\theta \left( \xi \right) $$ is enhanced by the rise in thermal radiation $$R_{1}$$ for both scenarios of inclination parameter $$\varpi $$ i.e., non-inclined MHD $$\left( \varpi =\pi /2\right) $$ and inclined MHD $$ \left( \varpi =\pi /4\right) $$ and also for electric parameter when $$ E_{1}=0.1,0.2$$. This is based on the fact that the increment in radiation parameter gives more heat to liquid which permitting the rise in temperature and thermal boundary layer thickness. Now, we elaborate the influence of electric parameter $$E_{1}$$ on the temperature field as it has also a strong physical significance on the fluid temperature. Figure 14 also demonstrates that there is high temperature and associated boundary layer thickness for increment in electric parameter $$E_{1}$$. Additionally, it is found that for both radiation and electric parameters the temperature field upsurges for the case of inclined MHD $$\left( \varpi =\pi /4\right) .$$ Figure 15 show the variability of temperature field for several values of heat source parameter $$Q_{1},$$ Biot number $$B_{i}$$ and thermal slip parameter $$\alpha $$ for both scenarios of inclination parameter $$\varpi $$ i.e., non-inclined MHD $$\left( \varpi =\pi /2\right) $$ and inclined MHD $$\left( \varpi =\pi /4\right) $$. It is noted that the temperature field rises with an increament of the heat source. Also similar trend is happening for higher values of Biot and thermal slip parameter $$\alpha $$ for both situations.

The values of SFC and NN for different values of related parameters are presented in Tables 3 and 4 for both inclined and non-inclined MHD by taking $\varpi =\pi /6,\pi /2$ respectively. It can be observed that SFC reduces with increment in Weissenberg number, unsteady number, velocity slip parameter, and suctions parameter $\left( A>0\right)$ while the opposite pattern is observed for local electric parameter, magnetic number and injection variable $\left( A<0\right)$ for both cases. It is examined that NN reduces with an increase in the magnetic parameter, Prandtl variable, thermal slip number and heat absorption parameter $\left( Q_{1}<0\right)$, whereas it grows with an increase in electric number, radiation number, Eckert parameter, Biot number and heat generation number $\left( Q_{1}>0\right)$ for both cases. The outcomes attained from the numerical modeling and ANN model are in quite strong agreement with the numerical outputs. The suggested ANN model is therefore effective for unsteady hydromagnetic Williamson liquid flow along the radiative surface via heat absorption/generation and convective boundary condition, based on results of the current study.

**Figure 16:**
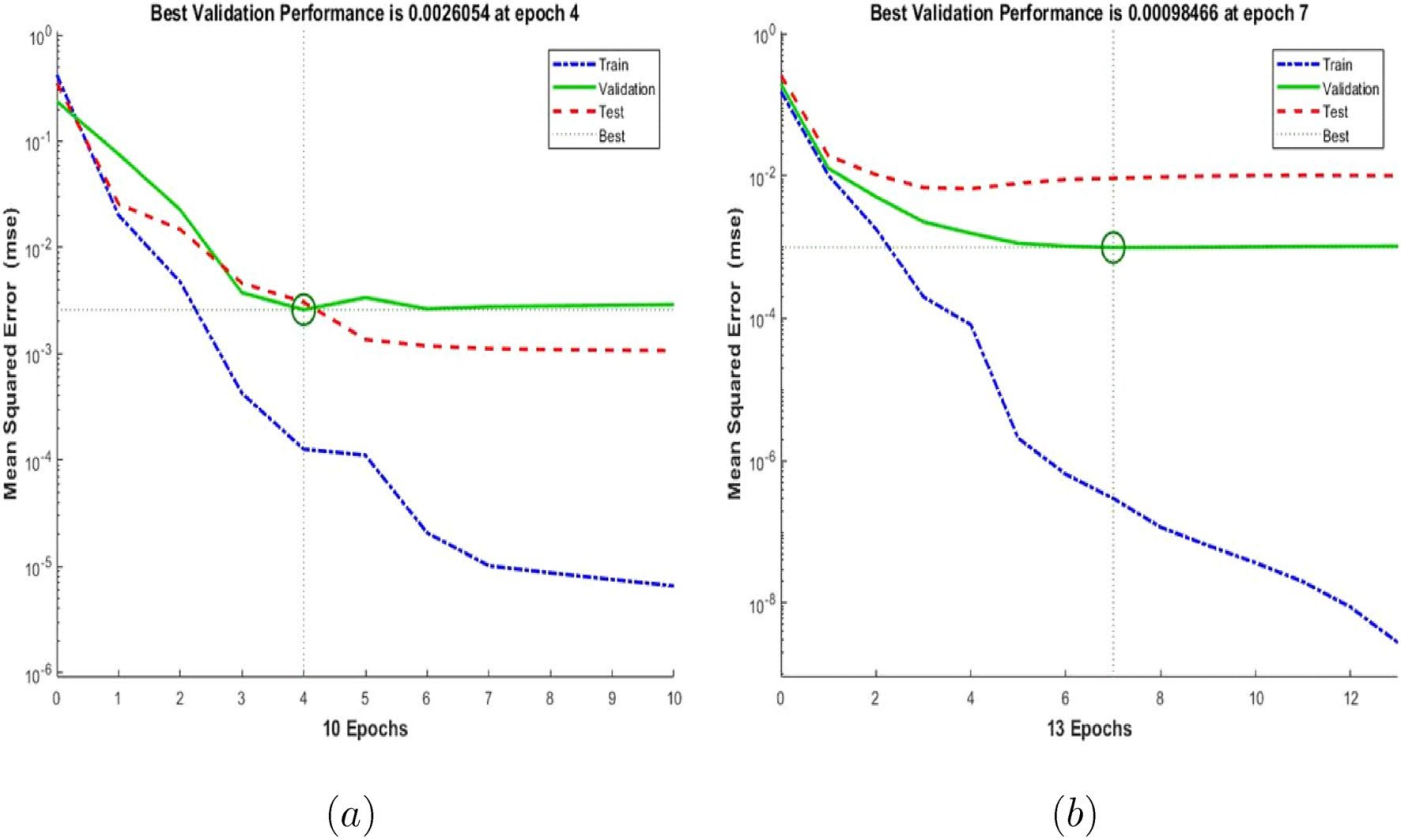
Performance of the ANN (**a**) Skin Friction Coefficients (**b**) Nusselt Number.

**Figure 17:**
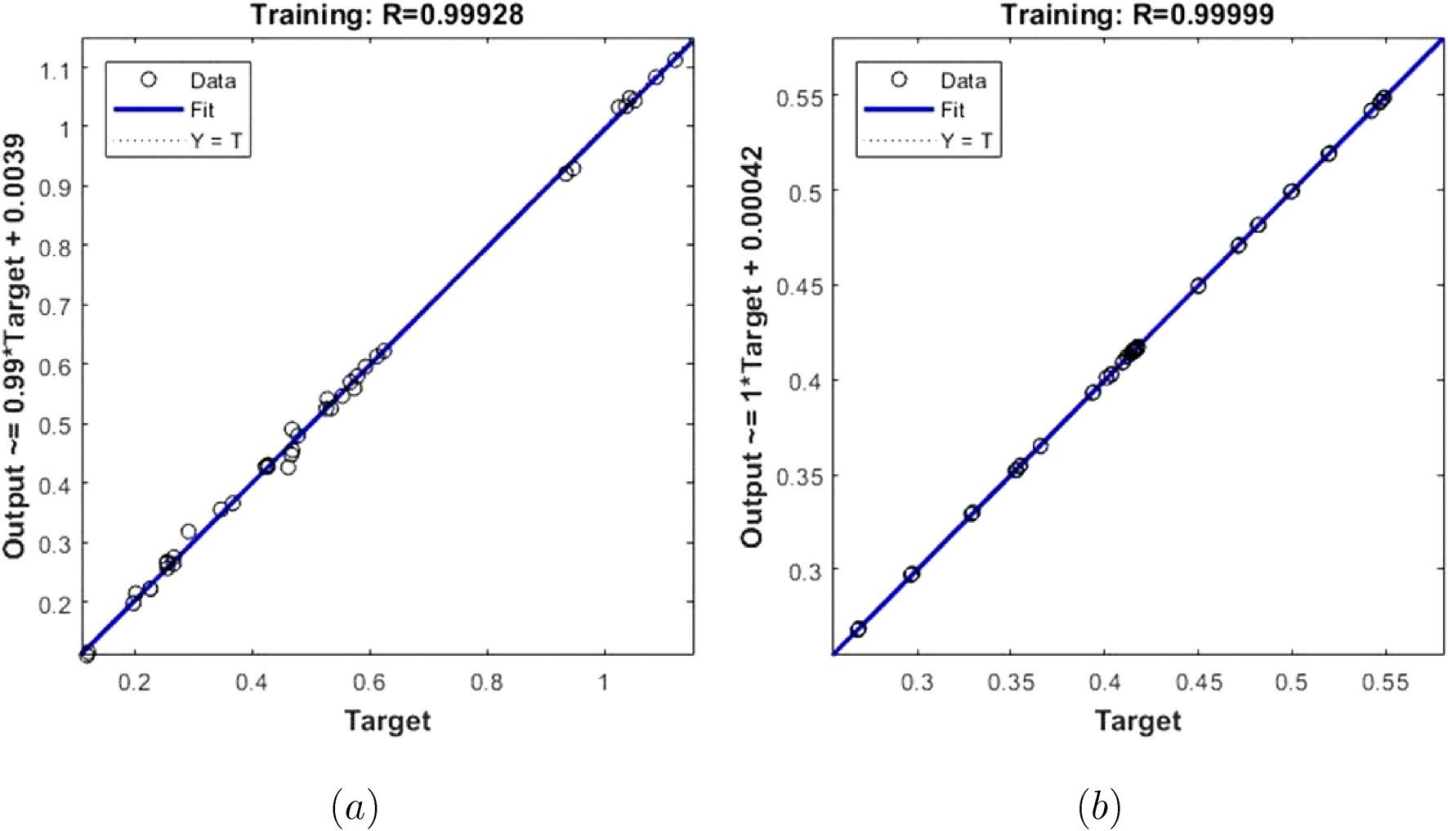
Training data (**a**) Skin Friction Coefficients (**b**) Nusselt Number.

**Figure 18:**
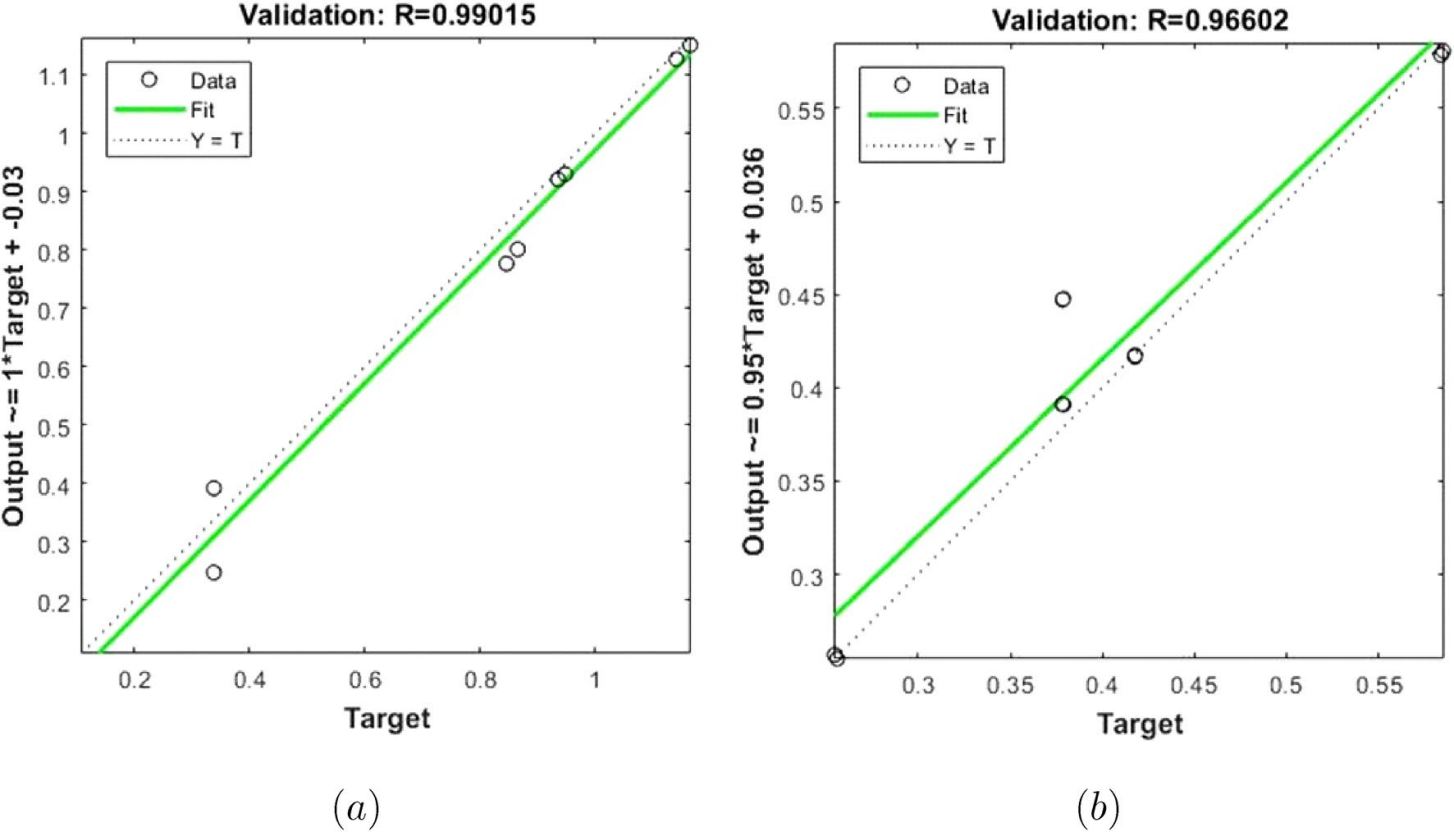
Validation data (**a**) Skin Friction Coefficients (**b**) Nusselt Number.

**Figure 19:**
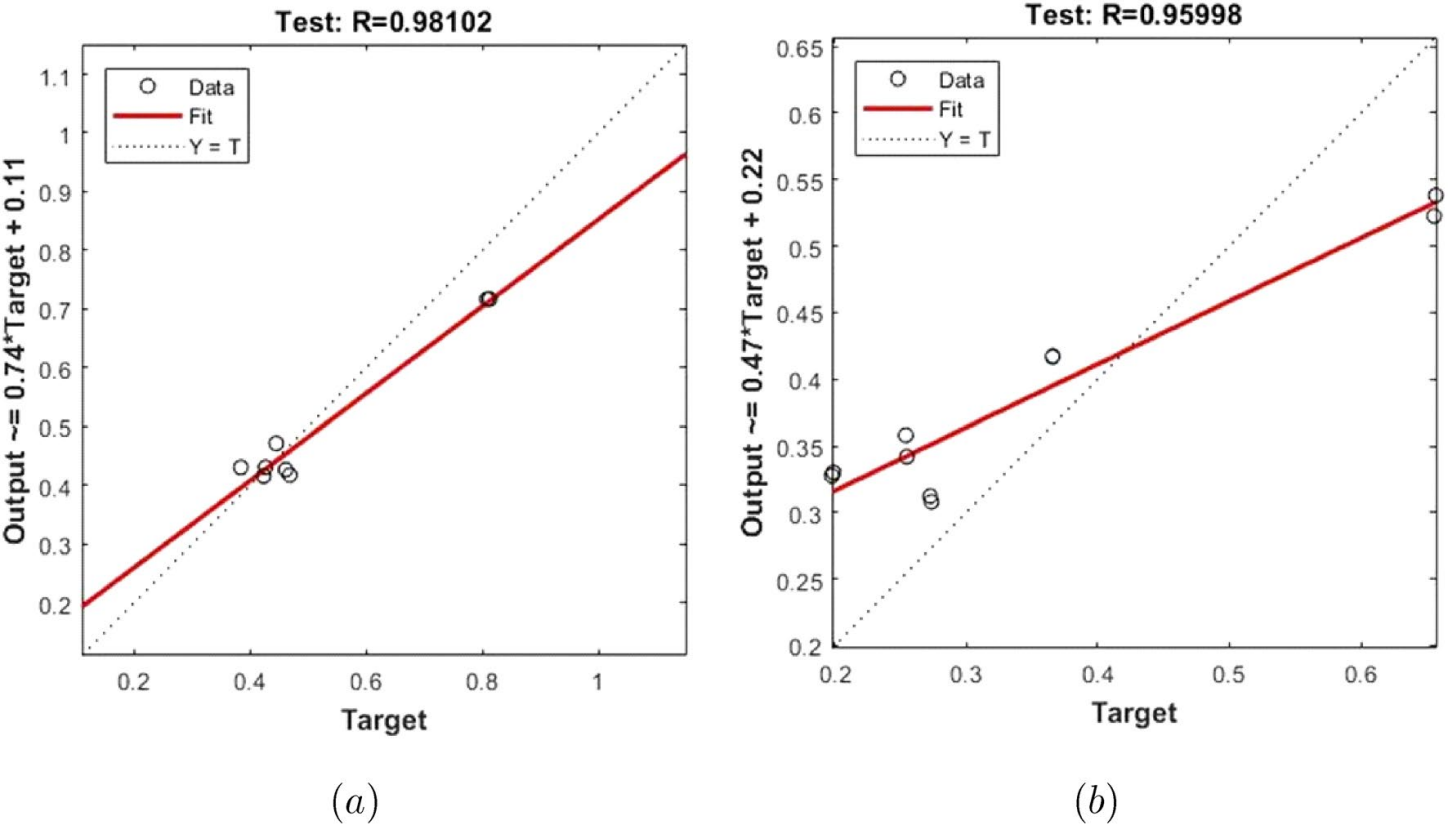
Test data (**a**)Skin Friction Coefficients (**b**) Nusselt Number.

**Figure 20:**
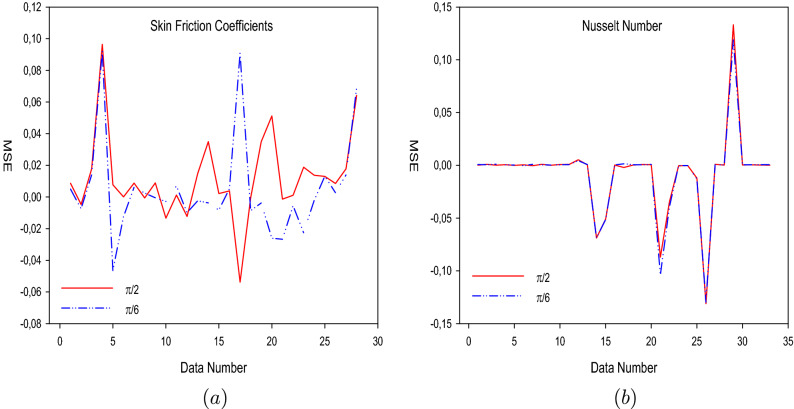
MSE values (**a**) Skin Friction Coefficients (**b**) Nusselt Number.

**Figure 21:**
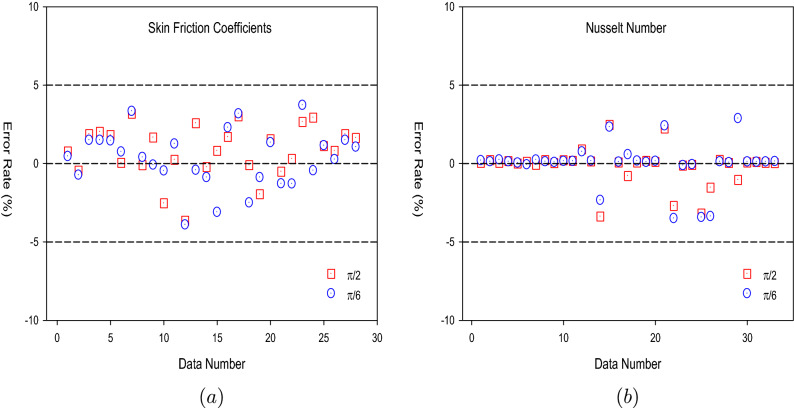
Error rates (**a**) Skin Friction Coefficients (**b**) Nusselt Number.

**Figure 22:**
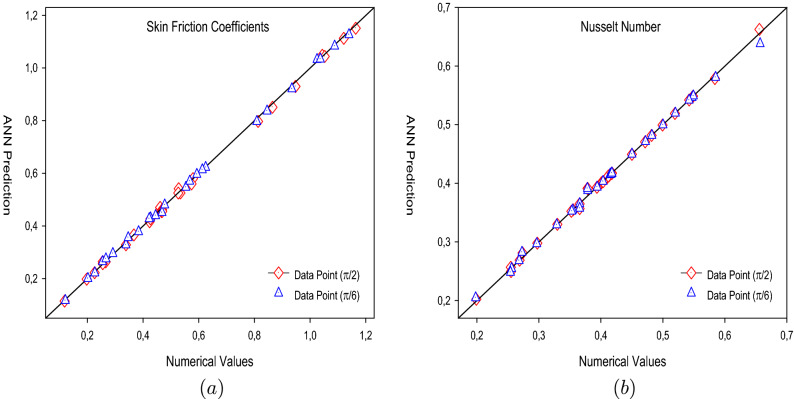
Numerical values vs ANN Prediction (**a**) Skin Friction Coefficients (**b**) Nusselt Number.

The training performances of both ANN models developed in Fig. 16 are shown. It is clear from the graphs that the MSE values, which have high values at the beginning of the training process, decrease with the advancing epochs. The ANN model, which has been developed to predict the Skin Friction Coefficients value, reached the best point by reaching the lowest MSE value in the 4th epoch. The ANN model developed to predict the Nusselt Number reached the lowest MSE value in the 7th epoch. This situation indicates that the training phase of ANN models has been completed with high performance. Figure 17 shows the data obtained from the training phase of ANN models. While there are target values on the x-axis of the graphs, there are ANN predictions on the y-axis. When the location of the data points obtained from the data used in the training phase of both developed models is examined, it is seen that they are located on the equality line drawn in blue. The R value for the ANN model developed for the Skin Friction Coefficients prediction is 0.99928 and the R value for the ANN model developed for the prediction of the Nusselt Number is calculated as 0.99999. These values show that the training phase of both ANN models has been ideally completed. Figure 18 shows the performance of the validation stage of both ANN models. When the graph is examined, it is seen that the data points obtained from the validation stage are close to the equality line drawn in green. However, R values for the models have been calculated as 0.99015 and 0.96602, respectively. The data obtained confirm that the validation stage of both ANN models has been completed with low error rates. In Fig. 19, the test stages of both ANN models is shown. It should be noted that the data points obtained from the test stages in the graphics are located close to the equality line drawn in red. R values of ANN models have been obtained as 0.98102 and 0.95998, respectively. These results clearly show that the test stages of ANN models have been completed with high accuracy. Figure 20 shows the MSE values obtained for each data point of the ANN models developed. While there are data points on the x-axis of the charts, there are MSE values on the y-axis. It should be noted that the values obtained by the calculated MSE values are quite low. The low values reached by the MSE values are another indication that both ANN models are ideally developed. In analyzing the performance of ANNs, it is important to analyze the error rates between predicted values and target values. For this purpose, error rates have been calculated and analyzed for each data point. In Fig. 21 the error rates of both ANN models are shown for each data point. When the graphics are examined, it is seen that the error rates of both models are low. When the error rates are evaluated, it is seen that the developed ANN models can predict Skin Friction Coefficients and Nusselt Numbers with acceptable error rates. Figure 22 shows the comparison of the prediction data obtained from both ANN models with the numerical data which are the target values. While there are target data on the x-axes of the graphs, there are ANN predictions on the x-axes. When the graphs are examined, it is seen that the data points obtained from both ANN models are located on the equality line. This state of the data points confirms that both ANN models are developed to be able to predict with high accuracy. Numerical values of performance parameters of both ANN models are given in Table 2.

**Table 1:** Arguments $x_{k}$ and corresponding coefficients $A_{k}$.

$x_{k}$	$B_{k}$
0.137793	0.308441116
0.729455	0.401119929
1.80834	0.218068288
3.40143	0.062087456
5.5525	0.009501517
8.33015	0.000753008
11.8438	0.000028259
16.2793	$4.24931 \times 10^{-7}$
21.9966	$1.83956 \times 10^{-9}$
29.9207	$9.91183 \times 10^{-13}$

**Table 2:** Numerical values of performance parameters of ANN models.

	Skin Friction Coefficients			Nusselt Number		
	MSE	R	MoD	MSE	R	MoD
Train	$1.27 \times 10^{-04}$	0.99928	0.27	$2.95 \times 10^{-07}$	0.99999	0.09
Validation	$2.61 \times 10^{-03}$	0.99015	1.82	$9.85 \times 10^{-04}$	0.96602	$-$1.06
Test	$3.07 \times 10^{-03}$	0.98102	0.86	$9.22 \times 10^{-03}$	0.95998	0.02
All	$1.93 \times 10^{-03}$	0.99540	0.57	$3.40 \times 10^{-03}$	0.93413	$-$0.10

**Table 3:** Numerical values of skin friction coefficients ${\text {Re}}_{{\hat{x}}}^{\frac{1}{2}}{\tilde{C}}_{F}$ for different values of physical parameters.

$W_{e}$	*A*	$S_{1}$	$M_{1}$	$E_{1}$	$\gamma _{1}$	$-{\text {Re}}_{{\hat{x}}}^{\frac{1}{2}}{\tilde{C}}_{F}$			
						GWRM		ANN Prediction	
						$\varpi =\pi /2$	$\varpi =\pi /6$	$\varpi =\pi /2$	$\varpi =\pi /6$
0.0	0.2	0.2	0.2	0.2	0.2	1.121120	1.088250	1.112467812	1.083272461
0.1						1.043753	1.024818	1.048470245	1.032320827
0.2						0.947400	0.934794	0.929619336	0.920865489
0.3						0.813207	0.808549	0.796758184	0.79651443
0.4						0.423486	0.383934	0.415797181	0.378361069
0.5						0.197690	0.201845	0.197602484	0.200345792
0.6						0.118298	0.120898	0.114536525	0.11686413
0.4	$-$0.3	0.2	0.2	0.3	0.2	0.579218	0.624945	0.579849492	0.622467226
	$-$0.1					0.534282	0.612793	0.525415549	0.613342799
	0.0					0.528116	0.593290	0.541500147	0.59608308
	0.1					0.526469	0.553815	0.525230251	0.54687993
	0.3					0.255066	0.254418	0.264369837	0.264359103
0.4	0.2	0.0	0.2	0.3	0.2	0.574345	0.567663	0.559570812	0.570079188
		0.2				0.461392	0.426647	0.462441292	0.430436492
		0.4				0.265973	0.266549	0.263805884	0.274831381
		0.6				0.226133	0.226746	0.222296971	0.221554163
0.4	0.2	0.2	0.0	0.3	0.2	0.338589	0.338589	0.328381059	0.327822824
			0.1			0.426647	0.346803	0.427074483	0.355467855
			0.2			0.461392	0.426647	0.470441292	0.430436492
			0.3			0.468562	0.445113	0.461320839	0.439161544
0.4	0.2	0.2	0.3	0.0	0.2	0.255378	0.291616	0.256710308	0.295370046
				0.2		0.367085	0.422143	0.365989456	0.427634432
				0.4		0.466563	0.468116	0.454175184	0.450696484
				0.6		0.468732	0.477804	0.455011903	0.479954302
0.2	0.2	0.2	0.2	0.2	0.0	1.163880	1.139770	1.150888953	1.126683758
					0.1	1.052630	1.036790	1.044065726	1.034083472
					0.2	0.947401	0.934794	0.929619336	0.920865489
					0.3	0.864947	0.845393	0.850830626	0.836475263

**Table 4:** Numerical values of Nusselt number $Re_{x}^{-1/2}Nu_{x}$ for different values of physical parameters $A=0.3,$ $S_{1}=0.0.2,W_{e}=0.4, \gamma _{1}=0.2$.

$M_{1}$	$E_{1}$	$R_{1}$	$P_{r}$	$E_{c}$	$Q_{1}$	$B_{i}$	$\alpha$	$-{\text {Re}}_{x}^{-1/2}Nu_{{\hat{x}}}$			
								GWRM		ANN Prediction	
								$\varpi =\pi /2$	$\varpi =\pi /6$	$\varpi =\pi /2$	$\varpi =\pi /6$
0.0	0.3	0.3	1	0.5	0.5	0.2	0.2	0.417812	0.417812	0.417622065	0.417011494
0.2								0.417615	0.417798	0.416708272	0.417312171
0.4								0.413635	0.417615	0.413437193	0.416625828
0.6								0.409676	0.416479	0.409060414	0.415986525
0.2	0.0	0.3	1	0.5	0.5	0.2	0.2	0.401078	0.414855	0.401080763	0.414687452
	0.1							0.409927	0.415234	0.409457697	0.415549836
	0.2							0.411936	0.416209	0.412264956	0.415243645
	0.3							0.417615	0.417798	0.416708272	0.417312171
0.2	0.3	0.0	1	0.5	0.5	0.2	0.2	0.268871	0.268210	0.268799609	0.268001409
		0.2						0.365731	0.365844	0.364971655	0.365299625
		0.4						0.471390	0.471922	0.47056126	0.471221267
		0.6						0.583913	0.585162	0.578643641	0.580752337
0.2	0.3	0.3	0.8	0.5	0.5	0.2	0.2	0.393609	0.394120	0.393078442	0.393476331
			0.9					0.378502	0.378705	0.391306506	0.387579291
			1.0					0.365730	0.365836	0.356708272	0.357312171
			1.1					0.355096	0.354673	0.35485935	0.354320921
0.2	0.3	0.3	1.0	0.0	0.5	0.2	0.2	0.254696	0.256038	0.256716643	0.254569521
				0.3				0.352399	0.353061	0.352157083	0.352463088
				0.6				0.450119	0.450186	0.449328272	0.449794601
				0.9				0.547505	0.547101	0.546883625	0.546264445
0.2	0.3	0.3	1.0	0.5	$-$0.4	0.2	0.2	0.255003	0.254178	0.249302396	0.248048876
					$-$0.2			0.273659	0.272779	0.281062975	0.282322934
					0.0			0.297440	0.296601	0.297826133	0.296945579
					0.2			0.329769	0.328813	0.330039994	0.329025368
					0.4			0.378853	0.378246	0.39093441	0.391221193
0.2	0.3	0.3	1.0	0.5	0.5	0.0	0.3	0.199100	0.197921	0.20215141	0.204587407
						0.2		0.403670	0.403598	0.402736781	0.403118255
						0.4		0.548724	0.549310	0.548508547	0.548963569
						0.6		0.655744	0.656985	0.662586879	0.638124434
0.2	0.3	0.3	1.0	0.5	0.5	0.3	0.0	0.542491	0.542391	0.542129796	0.541764069
							0.1	0.51954	0.520165	0.519009327	0.519677028
							0.2	0.499513	0.500135	0.499251651	0.499576203
							0.3	0.481828	0.482317	0.481695049	0.481651657

## Final remarks

In current study we successfully utilized the ANN approach for prediction of electro-hydrodynamic BLSF of Williamson fluid towards a permeable stretched surface under heat generation/absorption and convective boundary condition. The data attained from the training, validation and testing stages of the proposed ANN model have been analyzed with numerical techniques and proved to have excellent prediction accuracy. The presented ANN model is reliable, effective and time saving as it requires less effort and provides quick results than numerical techniques. Furthermore, it is inferred that the developed ANN model may be regarded as an appropriate and effective approach for solving the heat transfer aspects with Newtonian/non-Newtonian fluid flow challenges. The main key points of this study as follows:

- The velocity profile is lessened for increment in both magnetic and Wessinberg numbers.
- Temperature field rises with an increament of the heat source while similar trend is noted for higher values of Biot and thermal slip parameter.
- Skin friction coefficient reduces with increasing values of Weissenberg parameter, unsteady parameter for both inclined and non-inclined MHD cases.
- For the ANN model developed to predict the Skin Friction Coefficients values, the MSE value is $1.93\times 10^{-3}$, the R value is 0.99540 and the average error rate is $0.57\%$.
- For the ANN model developed to predict the Nusselt Number, the MSE value has been calculated as $3.40\times 10^{-3}$, the R value as 0.93413 and the average error rate as $-0.10\%$.
- These results showed that the developed ANN models have been developed in such a way that they can calculate Skin Friction Coefficients and Nusselt Number values with very low error rates and high accuracy.
- The results obtained from the study revealed that ANNs are one of the ideal tools that can be used to predict the Skin Friction Coefficients and Nusselt Number values.

## Nomenclature

$${\hat{T}}$$: Fluid’s temperature
$${\hat{T}}_{w}$$: Wall’s temperature
${\tilde{K}}$: Fluid’s thermal conductivity
$$E_{c}$$: Eckert number
${\tilde{\sigma }}^{*}$: Stefan–Boltzmann constant
$$\hat{\text {v}},{\hat{u}}$$,: Velocity components
$${\hbox {P}}_{r}$$: Prandtl number
$\alpha$: thermal slip number
$$M_{1}^{2}$$: Magnetic parameter
$$R_{1}$$: Radiation parameter
$$W_{e}$$: Weissenberg number
$${\tilde{\Gamma }}$$: Time constant
${\tilde{U}}_{w}$: Variable stretching velocity
$${\tilde{c}}_{p}$$: Specific heat
$Q_{1}$: Heat generation parameter
$$B_{i}$$: Biot number
${\hat{T}}_{\infty }$: Ambient fluid’s temperature
$\gamma _{1}$: Velocity slip number
$E_{1}$: Local electric number
$A_{1}$: Suction/injection coefficient
$$S_{1}$$: Unsteadiness parameter
$${\tilde{R}}(x)$$: Residual function

## Greek symbols

${\tilde{q}}_{r}^{*}$: Radiative heat flux
$${\tilde{\rho }}_{0}^{*}$$: Fluid density
${\check{\sigma }}^{*}$: Stefan–Boltzmann constant
$$\nu $$: Kinematic viscosity
$\varpi$: Angel of inclination
${\check{k}}_{1}$: Mean absorption coefficient
${\tilde{\rho }} _{0}^{*}$: Density of fluid

## Abbreviations

MLP: Multi-layer perceptron
ANN: Artificial neural network
GWRM: Galerkin weighted residual method
GLF: Gauss–Laguerre formula
FF: Feed forward
BP: Back propagation
MSE: Mean square error
FFBP: Feed-forward back-propagation
